# *miR-634* restores drug sensitivity in resistant ovarian cancer cells by targeting the Ras-MAPK pathway

**DOI:** 10.1186/s12943-015-0464-4

**Published:** 2015-11-17

**Authors:** Marijn T. M. van Jaarsveld, Patricia F. van Kuijk, Antonius W. M. Boersma, Jozien Helleman, Wilfred F. van IJcken, Ron H. J. Mathijssen, Joris Pothof, Els M. J. J. Berns, Jaap Verweij, Erik A. C. Wiemer

**Affiliations:** Department of Medical Oncology, Erasmus MC Cancer Institute, Erasmus University Medical Center Rotterdam, Wytemaweg 80, 3015 CN, Rotterdam, The Netherlands; Erasmus Center for Biomics, Erasmus University Medical Center, 3015 CN, Rotterdam, The Netherlands; Department of Genetics, Erasmus University Medical Center, 3015 CN, Rotterdam, The Netherlands; Present address: Max Planck Institute for Molecular Genetics, Berlin, Germany

**Keywords:** microRNA, miRNA, Drug resistance, *miR-634*, Ras-MAPK pathway, Ovarian cancer, Chemotherapy, Cisplatin, RPS6KA3, RSK2

## Abstract

**Background:**

Drug resistance hampers the efficient treatment of malignancies, including advanced stage ovarian cancer, which has a 5-year survival rate of only 30 %. The molecular processes underlying resistance have been extensively studied, however, not much is known about the involvement of microRNAs.

**Methods:**

Differentially expressed microRNAs between cisplatin sensitive and resistant cancer cell line pairs were determined using microarrays. Mimics were used to study the role of microRNAs in drug sensitivity of ovarian cancer cell lines and patient derived tumor cells. Luciferase reporter constructs were used to establish regulation of target genes by microRNAs.

**Results:**

*MiR-634* downregulation was associated with cisplatin resistance. Overexpression of *miR-634* affected cell cycle progression and enhanced apoptosis in ovarian cancer cells. *miR-634* resensitized resistant ovarian cancer cell lines and patient derived drug resistant tumor cells to cisplatin. Similarly, *miR-634* enhanced the response to carboplatin and doxorubicin, but not to paclitaxel. The cell cycle regulator CCND1, and Ras-MAPK pathway components GRB2, ERK2 and RSK2 were directly repressed by *miR-634* overexpression. Repression of the Ras-MAPK pathway using a MEK inhibitor phenocopied the *miR-634* effects on viability and chemosensitivity.

**Conclusion:**

*miR-634* levels determine chemosensitivity in ovarian cancer cells. We identify *miR-634* as a therapeutic candidate to resensitize chemotherapy resistant ovarian tumors.

**Electronic supplementary material:**

The online version of this article (doi:10.1186/s12943-015-0464-4) contains supplementary material, which is available to authorized users.

## Background

Ovarian tumors are a group of molecularly and etiologically heterogeneous cancers [1], and are the fifth most common cause of cancer related mortality among women. The current standard of therapy is debulking surgery and combination chemotherapy, consisting of a taxane (e.g. paclitaxel) and a platinum-based compound (e.g cisplatin or carboplatin) [2, 3]. Despite an initial high chemo-responsiveness, with response rates over 80 % [4], most advanced epithelial ovarian cancer patients will relapse and ultimately die of drug-resistant disease [5].

In the cellular response to cytotoxic substances, cell signaling pathways, such as the MAPK pathways, play a pivotal role. The classical MAPK pathway is activated by receptor tyrosine kinases (RTK), which bind GRB2 and SOS, after which the kinases RAS, RAF, MEK and MAPK are sequentially activated. Three major MAPK routes have been identified: the p38 MAPK, Jun kinase and ERK pathway. The MAPK pathways regulate numerous targets, including p90 ribosomal S6 protein kinases (RSKs), which in turn can activate downstream proteins. Together, MAPK pathways regulate the activity of genes involved in cell proliferation, DNA damage repair, cell cycle progression and apoptosis [6–10].

Although the mechanisms behind chemotherapy resistance in ovarian cancer have been studied extensively, the involvement of microRNAs (miRNAs), small RNAs that regulate gene expression, is just beginning to be unraveled [11, 12]. We have recently shown that *miR-141* targets *KEAP1* in ovarian cancer cells, repression of which results in enhanced cisplatin resistance [13]. In the current study we aimed to identify additional miRNAs that play a role in cisplatin resistance. Here, we describe that *miR-634* can sensitize both ovarian cancer cell lines and primary ovarian cancer cell cultures to chemotherapy. We show that *miR-634* regulates cyclin D1 and several Ras-MAPK pathway components (GRB2, ERK2, RSK1 and RSK2), which may contribute to the effects of *miR-634* on ovarian cancer cell survival and chemotherapy response.

## Results

### Comparison of miRNA expression profiles of cisplatin sensitive and resistant cell line pairs

In order to find miRNAs that play a role in cisplatin resistance, we compared miRNA expression profiles of cisplatin sensitive/resistant cell line pairs (IC_50_ values in Additional file 1: Table S1A). We hypothesized that in different cell types the same miRNAs play a role in cisplatin sensitivity, as has been reported for other factors involved in drug resistance [14]. Therefore, the miRNA expression pattern of an ovarian cancer cell line pair (A2780/A2780 DDP) was compared with expression patterns of a bladder cancer (T24/T24 DDP) and colon cancer (HCT8/HCT8 DDP) cell line pair. The only miRNA that showed a common pattern in all cell lines was *miR-634* (Additional file 1: Figure S1, FDR = 0.000), which was downregulated ≥1.5 fold in all cisplatin resistant cell lines (Additional file 1: Table S2). We further investigated the role of *miR-634* in ovarian cancer.

### Effects of miR-634 overexpression on cell cycle and apoptosis

**Fig. 1:**
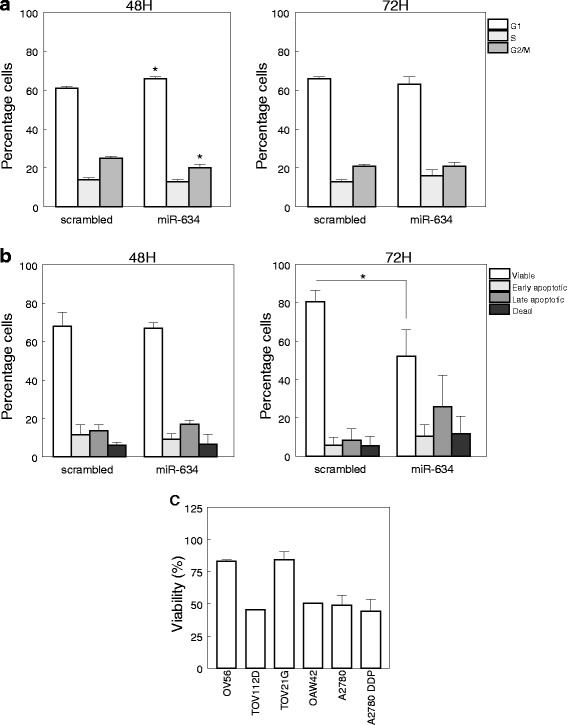
*MiR-634* overexpression induces G1 arrest and causes cell death. **a** Percentage of A2780 DDP cells in G0/G1, S or G2/M phase 48 or 72 h after transfection with a *miR-634* mimic or a scrambled control (*n* = 3), * = *p* < 0.05. **b** A2780 DDP cells were stained with PI and Annexin V 48 or 72 h after transfection with *miR-634* mimic or a scrambled control. Depicted are viable (PI/Annexin V negative), early apoptotic (Annexin V positive/PI negative), late (Annexin V positive/PI positive) and dead (PI positive/Annexin V negative) cells (*n* = 3). **c** Viability of *miR-634* mimic transfected ovarian cancer cells compared to cells transfected with a scrambled mimic (set at 100 %), as determined by an MTT assay 72 h after transfection. Depicted are average values ± SD (*n* = 3)

Before examining the effects of *miR-634* on cisplatin sensitivity, we determined whether *miR-634* overexpression affects the cell cycle and cell survival of A2780 DDP cells, which have a low basal *miR-634* expression compared to the parental A2780 cells. Upon transfection of the *miR-634* mimic, a slightly higher percentage of cells was observed in the G1 phase (*p* = 0.04) accompanied by a lower number of cells in the G2/M phase (*p* = 0.04) (Fig. 1a). These effects, which were observed at 48 h after transfection in multiple experiments, suggest that *miR-634* overexpression may affect the G1-to-S phase transition. At 72 h after transfection, however, the cell cycle profile of *miR-634* overexpressing cells was comparable to cells transfected with scrambled mimic (Fig. 1a).

Next, we examined if *miR-634* overexpression induces apoptosis. Whereas at 48 h after transfection the viability of control and *miR-634* mimic transfected cells was comparable, at 72 h the percentage of viable cells was significantly lower (*p* = 0.03) in *miR-634* transfectants, corresponding to increased numbers of apoptotic and dead cells (Fig. 1b). This effect of *miR-634* on apoptosis was also detected by MTT assay in five other ovarian cancer cell lines, A2780 (parental line), OV56, OAW42, TOV21G and TOV112D. In these cells *miR-634* gave rise to a 20–50 % reduction in viability, relative to control transfectants (Fig. 1c).

### MiR-634 enhances cisplatin sensitivity of ovarian cancer cell lines

**Fig. 2:**
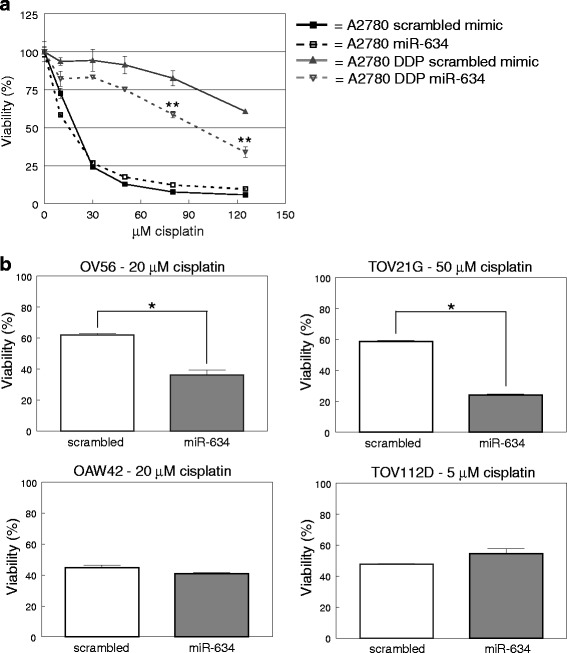
*MiR-634* mimic enhances sensitivity for cisplatin in ovarian cancer cell lines. Ovarian cancer cell lines were transfected with *miR-634* mimic or a scrambled control. 48 h after transfection, cisplatin was added and after 24H cell viability was determined with an MTT assay. **a** Overexpression of *miR-634* miRNA in the sensitive A2780 and resistant A2780 DDP cell lines. Error bars represent the standard deviation within one experiment (*n* = 5). ** = *p* < 0.01. **b** Overexpression of *miR-634* miRNA in the OV56, TOV21G, OAW42, TOV112D ovarian cancer cell lines. The cisplatin concentration is based on the IC_25_ value of mock transfected cells). Error bars represent the standard deviation within one experiment (*n* = 3). * = *p* < 0.05

We next determined the effects of *miR-634* overexpression on cisplatin sensitivity using a previously developed assay [13]. Briefly, cells were transfected with a *miR-634* mimic or a scrambled control, and after 48 h exposed to various concentrations of cisplatin. After another 24 h cell viability was determined using an MTT assay. Because miRNA transfection was transient we used 24 h drug exposure intervals. Note that the difference in IC_50_ values observed between drug sensitive and resistant cell lines was similar to IC_50_ values determined in assays with longer drug exposure times [13] (Additional file 1: Table S1A, C). As is shown in Fig. 2a, transfection with *miR-634* mimic gave rise to a marked increase in sensitivity after treatment with 80 μM (*p* = 0.006) and 125 μM cisplatin (*p* = 0.002) in the A2780 DDP cell line. A possible explanation for this sensitization may be a higher intracellular cisplatin accumulation and concomitantly increased cytotoxic activity. In fact, cisplatin resistance is frequently accompanied by reduced intracellular cisplatin levels [15] (Additional file 1: Table S1B). We measured the intracellular platinum content of *miR-634* and scrambled control transfected cells after exposure to 80 and 125 μM cisplatin for 2 h, 6 h and 12 h. *MiR-634* transfection did not affect the platinum uptake by A2780 DDP cells (Additional file 1: Figure S2) ruling out that *miR-634* sensitizes ovarian cancer cells for cisplatin by increasing its uptake and/or reducing its efflux.

In contrast to the resistant A2780 DDP cell line, enhanced expression of *miR-634* in the sensitive A2780 cells did not alter the sensitivity for cisplatin. This might be due to the fact that *miR-634* levels are already high in A2780 cells relative to A2780 DDP. We next examined the effect of *miR-634* transfection on other ovarian cancer cell lines. Because the most significant effect was observed at the IC_25_ dose in the A2780 DDP cell line, the other ovarian cancer cell lines were treated with the IC_25_ dose of mock (transfection reagent only) transfected cells (Additional file 1: Table S1C). In two of the most resistant cell lines in this assay, OV56 and TOV21G, *miR-634* overexpression significantly reduced viability (Fig. 2b). In contrast, in the more sensitive cell lines OAW42 and TOV112D, overexpression of *miR-634* did not alter sensitivity. The effect of *miR-634* on cisplatin response thus appears to be strongest in resistant cell lines.

### MiR-634 sensitizes tumor cells from patients with drug resistant ovarian cancer to chemotherapy

Ovarian cancer is routinely treated with platinum (e.g. cisplatin, carboplatin) and taxane (e.g. paclitaxel) based combination chemotherapy. However, most tumors eventually become resistant. As *miR-634* overexpression can increase cisplatin sensitivity of cell lines, we examined whether this treatment could also sensitize chemotherapy resistant primary ovarian tumor cells. Ascites was collected from 6 patients with serous and 1 patient with clear cell ovarian cancer (for patient characteristics, see Table 1). One patient was chemotherapy naïve, the others had received carboplatin/paclitaxel combination regimens and were or had become resistant. The ovarian cancer cells were isolated and cultured from ascites as described previously [16, 17], All cultures tested positive for the epithelial marker pan-keratin (Additional file 1: Figure S3). Another epithelial marker, EpCAM, was only positive in tumor cells from patient 2, however it is known that primary ovarian cancer cells may lose EpCAM expression in culture [16]. To verify that the cultures contain tumor cells, p53 staining was analyzed as p53 is often mutated in high and intermediate grade serous ovarian cancer [18]. A clear nuclear p53 staining was observed in all cultures, indicating the cultures do indeed consist of tumor cells.

**Table 1:** Ovarian cancer patient characteristics

	Histological subtype	FIGO stage	Grade	Cytotoxic agents used	Response to last Pt containing treatment
Patient 1	Serous	IV	2	Carboplatin, Paclitaxel	PD within 1 month
Patient 2	Serous	IIIc	UK	Carboplatin, Paclitaxel, Gemcitabine	PD within 4 months
Patient 3	Serous	IIIc	3	Carboplatin, Paclitaxel, Gemcitabine	PD within 1 month
Patient 4	Serous	IIIc	2	Carboplatin, Paclitaxel, Olaparib, Caelyx	PD during treatment
Patient 5	Clear cell	IIIc	3	Carboplatin, Ifosfamide	PD within 1 month
Patient 6	Serous	IIIc	3	Carboplatin, Paclitaxel, Caelyx	PD within 5 months
Patient 7	Serous	IIIc	2		Chemotherapy naive

*UK:* Unknown, *Pt:* Platinum, *PD:* Progressive disease, Caelyx: liposomal doxorubicin

**Fig. 3:**
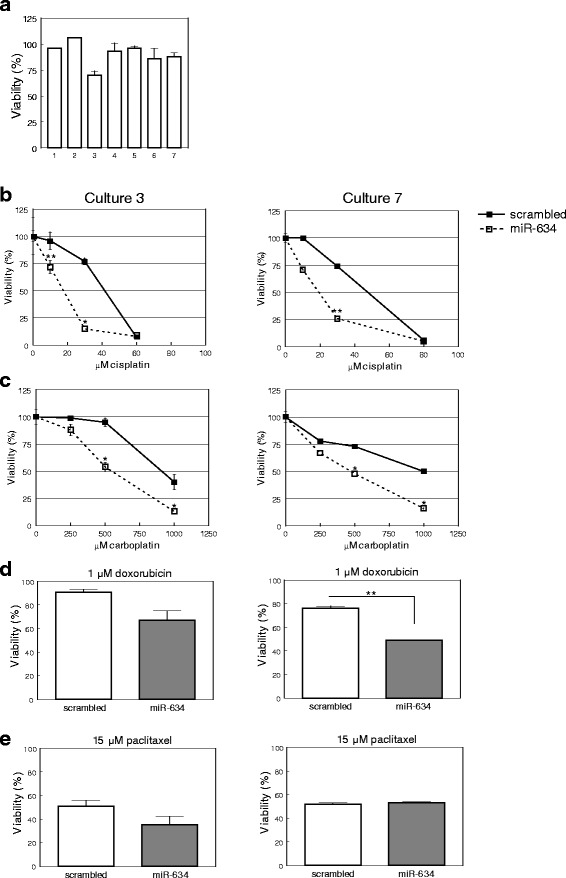
*MiR-634* mimic enhances chemotherapy sensitivity in primary ovarian cancer cell cultures. **a** Overexpression of *miR-634* in primary ovarian cancer cell cultures, derived from the ascites of ovarian cancer patients, has no or a moderate effect on cell viability 72 h after transfection. Indicated is the viability of *miR-634* transfected cells relative to the viability of cells transfected with scrambled mimic (set at 100 %). The numbers on the X-axis refer to the number of the culture. Depicted are average values ± SD (*n* = 3). **b** *MiR-634* enhances the sensitivity for cisplatin. Depicted are the results of a representative experiment for culture 3 (left, *n* = 4) and culture 7 (right, *n* = 3). An overview of the results obtained for all cultures is in Additional file 1 Figure S4. * = *p* < 0.05, ** = *p* < 0.01. **c** *MiR-634* enhances the sensitivity for carboplatin. Depicted are the results of a representative experiment for culture 3 (left, *n* = 3) and culture 7 (right, *n* = 4). An overview of all cultures that were treated with carboplatin is in Additional file 1: Figure S5. * = *p* < 0.05, **d** *MiR-634* enhances the sensitivity for doxorubicin. Depicted are the results of a representative experiment for culture 3 (left, *n* = 3) and culture 7 (right, *n* = 3). ** = *p* < 0.01. **e** *MiR-634* does not significantly alter the sensitivity for paclitaxel. Depicted are the results of a representative experiment for culture 3 (left, *n* = 3) and culture 7 (right, *n* = 3)

We first tested whether *miR-634* overexpression in primary tumor cells gave rise to a reduced viability, as was observed for the ovarian cancer cell lines. Remarkably, *miR-634* overexpression in primary cell cultures only mildly diminished (5–25 %) the number of viable cells (Fig. 3a).

*miR-634* overexpression gave rise to a significant and reproducible decrease in cisplatin resistance in five cultures (Fig. 3b, Additional file 1: Figure S4). Note that in cultures 1 and 2 *miR-634* overexpression gave rise to a similar phenotype, however, they could only be tested once due to low cell numbers. When the data of all primary cultures were combined, *miR-634* overexpression was associated with a significant reduction in cellular viability of cells treated with 15 or 30 μM cisplatin (*p* = 0.002, *p* < 0.001, respectively).

Next, we tested if *miR-634* could alter the response towards other anticancer compounds. *miR-634* overexpression gave rise to significant increases in carboplatin sensitivity in cultures 3 and 7 (Fig. 3c, Additional file 1: Figure S5) as well as reduced resistance in two other cultures (which could only be tested once). We could not examine other cultures because of low cell numbers. In addition, *miR-634* overexpression gave rise to a significant increase in doxorubicin sensitivity (*p* = 0.004), whereas there was no significant reduction in sensitivity for paclitaxel (*p* = 0.335) (Fig. 3d, e). These data indicate that *miR-634* overexpression sensitizes resistant primary ovarian cancer cells to cisplatin, carboplatin and doxorubicin.

### MiR-634 modulates the expression of key proliferation genes

**Fig. 4:**
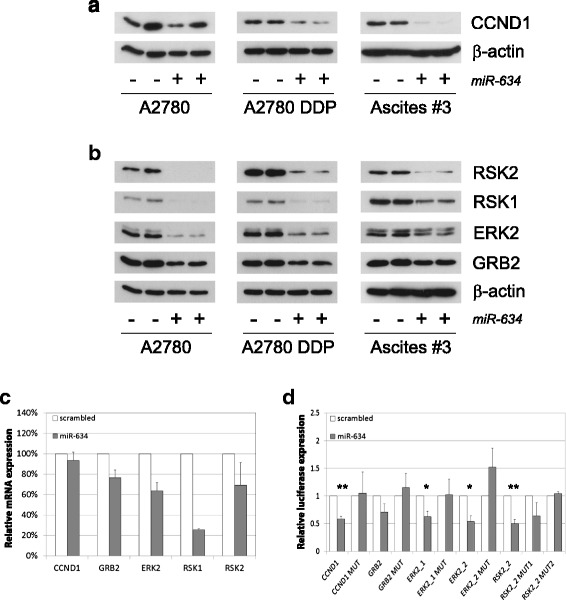
*MiR-634* regulates genes involved in cell cycle regulation and the Ras-MAPK pathway. Ovarian cancer cell lines (A2780, A2780 DDP) and patient derived tumor cells (culture #3) were transiently transfected with scrambled control (-) or *miR-634* mimic (+). 48 h after transfection protein lysates were analyzed for the expression of putative *miR-634* target genes by Western blotting (A, B) or RT-PCR (C). **a** Transfection with *miR-634* reduces Cyclin D1 protein levels in the A2780 cell lines and in primary ovarian cancer cell cultures. β-actin is used as a loading control. **b** *MiR-634* overexpression reduces protein levels of GRB2, ERK2, RSK1 and RSK2, components of the Ras-MAPK pathway in the A2780 and A2780 DDP cell lines and primary ovarian cancer cell cultures. β-actin is used as a loading control. **c** *MiR-634* overexpression lowers mRNA levels of CCND1, GRB2, ERK2, RSK1 and RSK2 in the A2780 DDP cell lines 48 h after transfection. Expression was normalized to HPRT and GAPDH expression. Depicted are average values ± SD (*n* = 2). The mRNA level in cells transfected with scrambled mimic is set at 100 %. **d** *MiR-634* binds directly to elements of the 3’UTR of CCND1, GRB2, ERK2 and RSK2. A2780 DDP cells were transfected with a scrambled mimic or a *miR-634* mimic. After 8 h, the same cells were transfected with a *Renilla* luciferase reporter construct (psiCHECK™-2) containing a region of 500 bp surrounding the predicted target sites (Additional file 1: Figure S7), or a luciferase construct in which the *miR-634* binding sites were mutated. Since RSK2 contains both a canonical and a non-canonical *miR-634* binding site, one construct was generated that contained a mutation in the canonical binding site (RSK2_2 mut 1) and another construct that contained mutations in both the canonical and non-canonical site (RSK2_2 mut 2). The Renilla luciferase activity was measured and normalized using the Firefly luciferase activity. For each construct, the relative luciferase activity of cells transfected with the scrambled mimic was set at 1. Depicted are the average values ± SD (*n* = 3). * *p* < 0.05, ** *p* < 0.01

MiRNAs mainly act by downregulating the expression of protein-coding genes. In order to identify target genes, we performed a pathway analysis on genes predicted to be a *miR-634* target. Among the most enriched Gene Ontology (GO) terms were ‘positive regulation of cyclin-dependent protein kinase activity’ and ‘regulation of the G1-S transition of the mitotic cell cycle’ (Additional file 1: Figure S6, Table S3). Notably, Cyclin D isoforms CCND1 and CCND2 were associated with these GO terms, which activity is required for the G1 to S phase transition. Overexpression of *miR-634* resulted in a decreased CCND1 protein level in A2780, A2780 DDP and primary tumor cells (derived from patient 3) (Fig. 4a) and this may explain the slight increase in G1 cells at 48 h after transfection (Fig. 1a).

Another GO term that was enriched was ‘positive regulation of proliferation’. Several proliferation pathways were predicted to be regulated by *miR-634*, most notably the Ras-MAPK pathway, of which *GRB2*, *N-RAS*, *RAF*, *ERK2*, *RSK1* and *RSK2* (Additional file 1: Table S3 and S4, Additional file 1: Figure S6) are potential targets. Concurringly, overexpression of *miR-634* gave rise to reduced protein levels of GRB2, ERK2, RSK1 and RSK2 in the A2780 cell lines and primary cell culture 3 (Fig. 4b). Since miRNAs can regulate target expression by translational repression and/or mRNA degradation, we also examined target mRNA levels. Whereas there was only a slight decrease in *CCND1* mRNA levels in *miR-634* transfected cells, we observed a 25–75 % decrease in *GRB2*, *ERK2*, *RSK1* and *RSK2* mRNA levels (Fig. 4c).

We next tested whether *miR-634* could directly regulate these genes. For each of the potential target genes, luciferase constructs were generated that express part of the 3’UTR containing the *miR-634* target site (Additional file 1: Figure S7). As *ERK2* and *RSK2* contain respectively three and two canonical *miR-634* target sites, the 3’UTR surrounding these sites was cloned separately. As expected, *miR-634* could inhibit the 3’UTR of *CCND1* and *GRB2*, the *ERK2* 3’UTR (constructs ERK2_1 and ERK2_2) and the *RSK2* 3’UTR (construct RSK2_2) (Additional file 1: Figure S8). In contrast, we observed no repression of the *miR-634* target site in the *RSK1* 3’UTR, suggesting that the effect of *miR-634* on *RSK1* protein and mRNA levels may be indirect.

To confirm specific binding, the *miR-634* binding site was mutated in the relevant constructs (CCND1, GRB2, ERK2_1, ERK2_2, RSK2_2). Since construct RSK2_2 contained two potential binding sites (Additional file 1: Figure S7), we created one construct with a mutation in the canonical site only (RSK2_2 mut 1) and another construct with mutations in both sites (RSK2_2 mut 2). As indicated in Fig. 4d, *miR-634* could no longer repress the mutated 3’UTRs of *CCND1*, *GRB2*, and *ERK2*. Interestingly, mutation of both sites in RSK2_2 was necessary to completely prevent *miR-634* binding.

As an alternative approach to inhibit *miR-634* function we assessed the effect of a specific antisense inhibitor on the luciferase activity of the wild-type 3’UTRs of *miR-634* target genes. As shown in Additional file 1: Figure S9, inhibition of endogenous *miR-634* led to increased luciferase activity with the RSK2_2 and, to a lesser extent, GRB2 constructs, indicating that endogenous *miR-634* regulates the expression of these target genes. In this setup we did not find an increased luciferase activity for the ERK2 or CCND1 constructs, and therefore we cannot confirm that ERK2 and CCND1 are endogenous *miR-634* targets in the A2780 DDP cell line. We next examined the relation between *miR-634* expression and expression levels of its target genes in a large cohort of ovarian serous cystadenoma’s (TCGA dataset) (Additional file 1: Figure S10). Unfortunately, in the majority of tumors *miR-634* expression was not detected above background. However, when we examined the 23 tumors with high *miR-634* expression (expression > mean + stdev) we found a significant negative correlation between *miR-634* levels and RSK2 (*p* = 0.002). We also found an inverse relation between *miR-634* and ERK2 (*p* = 0.091 or *p* = 0.310 for different probes), RSK1 (*p* = 0.372) and GRB2 (*p* = 0.614), but in this set of 23 expressing tumors this relation was not significant. It is possible that in a larger dataset this relation becomes significant. We did not observe an inverse relation between *miR-634* and CCND1 expression in this dataset.

Altogether, our findings show that overexpression of *miR-634* leads to direct repression of *RSK2*, *CCND1, GRB2* and *ERK2* in ovarian cancer cell lines and ovarian cancer cells derived from patients. Repression of the Ras-MAPK pathway may contribute to the decrease in cellular proliferation observed upon *miR-634* overexpression (Fig. 1b, c, d). In line with this, we observed that the MEK inhibitor PD0325901 reduced cellular viability at 48H after treatment (Additional file 1: Figure S11A). In addition, Ras-MAPK signaling may contribute to cisplatin resistance [19]. Our previous work has demonstrated that RSK2 depletion enhances cisplatin sensitivity [20]. Concurringly, PD0325901 augmented cisplatin toxicity at 24H after treatment (Additional file 1: Figure S11B).

## Discussion

The aim of this study was to discover miRNAs that affect the response of ovarian cancer cells to cisplatin chemotherapy. We compared the expression of three cisplatin-sensitive and –resistant cell line pairs and identified *miR-634* as a miRNA that can modulate the sensitivity to various drugs in ovarian cancer cells.

*miR-634* is located on chromosome 17 within intron 15 of *PRKCA* (Protein Kinase C α), and is only conserved in primates. *miR-634* has been first detected in colon cancer cells [21] via miRNA serial analysis of gene expression (miRAGE). Afterwards, *miR-634* has been identified as a miRNA able to regulate the expression of the androgen receptor (AR) in prostate cancer cells [22]. Repression of AR resulted in a reduced viability, however, the effect of *miR-634* overexpression was stronger than the effect of AR siRNA, suggesting that *miR-634* may target other survival genes as well. We now report that *miR-634* overexpression results in downregulation of multiple genes of the Ras-MAPK pathway, an important cell proliferation pathway that is activated in many types of cancer [8].

The observation that *miR-634* is able to regulate multiple Ras-MAPK pathway genes instead of one key mediator is thought-provoking. Interestingly, many miRNAs appear to synergistically regulate a set of genes that participate in similar processes, such as the *miR-17-92* cluster (regulates genes involved in growth control) [23], *let-7* (represses Ras and its downstream target HMGA2) [24, 25] and the *miR-200* family (represses the epithelial-to-mesenchymal transition (EMT) [26–29]. MiRNAs may thus allow cells to effectively switch off similar signaling pathways in response to changing circumstances, and this may be especially relevant for proliferative pathways, which activity may need to be tightly controlled. In addition, it may be advantageous for cells to switch off production of proteins for signaling pathways that are not active.

Repression of oncogene activity often leads to cell death in cancer cells, a phenomenon known as ‘oncogene addiction’ [30]. The reduction in cell viability observed in the ovarian cancer cell lines upon overexpression of *miR-634* may be caused by repression of the Ras-MAPK pathway. Interestingly, the effects of *miR-634* overexpression in primary cultures are much less pronounced, suggesting that these cells depend less on proliferation signaling. In support of this theory, the ascites derived tumor cells divided more slowly than the ovarian cancer cell lines, and the fastest growing cultures (cultures 3 and 7) showed the largest reduction in cell viability upon *miR-634* overexpression.

**Fig. 5:**
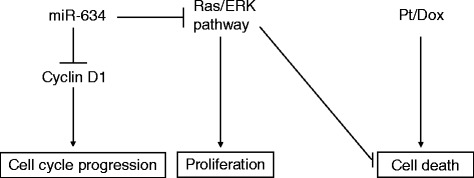
*MiR-634* enhances the sensitivity for chemotherapy. By regulation of Cyclin D1 and the Ras-MAPK pathway, *miR-634* overexpression may affect the cell cycle profile and apoptosis. Furthermore, repression of the Ras-MAPK pathway, which inhibits several pro-apoptotic factors (e.g. Bad [55], BimEL [56, 57]), can indirectly sensitize ovarian tumors to chemotherapeutics, such as cisplatin/carboplatin (Pt) and doxorubicin (dox)

We describe that *miR-634* transfection results in enhanced cisplatin sensitivity. Intriguingly, this effect of *miR-634* overexpression is most apparent in resistant ovarian cancer cell lines, and also occurs in tumor cells derived from ascites. The *miR-634* mediated repression of the Ras-MAPK pathway might contribute to the sensitization [19], which is supported by our finding that a MEK inhibitor enhances cisplatin sensitivity. Both ERK2 and RSK2 can inhibit several pro-apoptotic genes [31–35], and as a consequence of repression by *miR-634*, downregulation of ERK2 and RSK2 might lower the threshold for apoptosis upon treatment with cytotoxic therapy (Fig. 5). Indeed, our previous study shows that depletion of RSK2 leads to increased cisplatin sensitivity [20].

Because of the pivotal role of the Ras-MAPK pathway in cell proliferation and cancer, inhibitors have been developed that target this pathway [36]. However, the objective response rates are modest [37], perhaps because tumors may rewire their signaling pathways [38]. Since *miR-634* can inhibit several key proliferation factors simultaneously, acquired resistance is less likely to be a problem for a *miR-634* based therapy than it is for targeted agents. A next step would be to study the effect of *miR-634* overexpression on tumor growth and therapy response in a genetic mouse model for ovarian cancer. However, such a study is complicated by the fact that there is no high grade serous mouse model for ovarian cancer [39, 40], and *miR-634* has no murine orthologue.

Furthermore, *miR-634* levels in tumors may correlate with response to chemotherapy. Although some people have detected *miR-634* expression using miRAGE and microarrays [22, 41], these studies do not report quantitative PCR validation. In addition, we and a few others were unable to detect endogenous *miR-634* by RT-PCR [21, 42], despite evidence that the assay is functional (e.g. housekeepers RNU43, RNU48 were readily detected in our samples and we could find a *miR-634* signal in mimic-transfected cells; Additional file 1: Figure S12). However, some publications describe successful *miR-634* detection in RT-PCR assays [43–45]. An explanation for the discrepancy between studies could be that *miR-634* is modified under certain cell-type specific conditions. Of note, modifications at the 3’ end are not uncommon for microRNAs [46, 47] and may prevent *miR-634* detection by RT-PCR. Therefore, in order to monitor *miR-634* levels in tumors, a specific high throughput *miR-634* detection tool needs to be developed.

## Conclusions

In summary our data indicate that *miR-634* is an important player in cisplatin-resistance. First of all, *miR-634* was the only miRNA that was commonly downregulated in three cisplatin/sensitive cell line pairs. Overexpression of *miR-634* transiently inhibited G1-S cycle progression and enhanced apoptosis of ovarian cancer cells. Furthermore, *miR-634* enhanced the chemotherapy response of cisplatin-resistant ovarian cancer cell lines and drug resistant patient-derived primary tumor cells. In addition, we observed that *miR-634* overexpression in ovarian cancer cell lines and patient samples negatively regulates important cell-cycle genes (CCND1) and Ras-MAPK pathway components (GRB2, ERK2, RSK1 and RSK2). Inhibition of the Ras-MAPK pathway resulted in increased sensitivity to cisplatin, suggesting that the *miR-634*-mediated repression of this pathway is responsible for the effect of *miR-634* on cisplatin resistance. In the future, therapeutic delivery of this miRNA to drug resistant ovarian cancer cells may help to resensitize patients to treatment.

## Methods

### Cell culture and reagents

The ovarian carcinoma cell line A2780, colon carcinoma cell line HCT8, bladder carcinoma cell line T24 and their cisplatin-resistant derivatives A2870 DDP, HCT8 DDP, and T24 DDP10 have been described before [14, 15, 48, 49]. Resistant cell lines were routinely challenged with cisplatin. Ovarian cancer cell lines OV56 and OAW42 were purchased from the ECACC (Salisbury, UK) and TOV112D, TOV21G were obtained from the ATCC (Manassas, VA, USA). Characteristics of the ovarian cancer cells were published in [50] and the authenticity of cell lines was verified by STR analysis. All cell lines were cultured in RPMI 1640 Glutamax (Invitrogen, Bleiswijk, The Netherlands) supplemented with 10 % FBS (Greiner, Alphen a/d Rijn, The Netherlands) and Penicillin/Streptomycin (final concentration 100 IE each, Sigma, Zwijndrecht, The Netherlands). Cisplatin, Carboplatin, Paclitaxel and Doxorubicin were obtained from Pharmachemie, Haarlem, the Netherlands. PD0325901 (cat # PZ0162) was obtained from Sigma-Aldrich.

Isolation and culture of primary ovarian tumor cell cultures occurred as described before [16, 17]. Briefly, 25 mL ascites fluid was mixed 1:1 with MCDB105/M199 medium supplemented with 0.5 μg/mL Fungizone and 50 μg/mL Gentamycine (all obtained from Sigma-Aldrich), and incubated at 37 °C, 5 % CO_2_, in T75 flasks for 4 days. Afterwards, the medium was replaced with fresh MCDB105/M199 medium and replaced twice weekly until cells were confluent. Cells were split 1:2-1:3 for up to 6 passages. To verify that the cultures contain tumor cells, cytospins were analyzed for epithelial markers (pan-keratin and EpCAM) and p53.

The study was approved by the Medical Ethical Committee of the Erasmus University MC (MEC-2008-183).

### Microarray and data analysis

Total RNA was isolated with RNA Bee (BioConnect, Huissen, the Netherlands), and 1 μg RNA was labeled with the Cy3-TM ULS labeling kit (Kreatech Biotechnology, Amsterdam, The Netherlands), according to the manufacturer’s instructions. The RNA was hybridized with the LNA-based capture probe set (Exiqon, Vedbaek, Denmark) version 10 (annotation version 13). This probe set consists of 1344 probes including 725 human miRNAs, which are spotted in duplicate. Spots were quantified with the Imagene software (BioDiscovery), obvious outliers were removed and quantile normalization was performed.

miRNA profiling experiments were performed with RNA isolated at two (HCT8 and T24) or three (A2780) different passages. The average miRNA expression in the sensitive cell line was compared with the average expression in the resistant cell line and the fold-change was calculated. The microarray expression data has been deposited to the Gene Expression Omnibus (GEO) data repository (accession number GSE54665).

### RT-PCR

0.5-1 μg RNA was reverse transcribed using random primers (Applied Biosystems, Bleiswijk, The Netherlands). 45 ng cDNA was used in a real-time PCR reaction using Taqman® assays-on-demand (*CCND1*, *ERK2*, *GRB2*, *RSK1*, *RSK2*). Expression was normalized to *HPRT* and *GAPDH* expression using the comparative C_T_-method [51]. MiRNA expression was determined using Taqman® miRNA assays (Applied Biosystems) according to the manufacturer’s protocol. In brief, 50 ng of total RNA was reverse transcribed using specific miRNA primers. The cDNA was used as input in a quantitative real-time PCR. RNU43 and RNU48 expression were used for normalization using the comparative C_T_- method [51].

### Transfection

A miRIDIAN mimic for *miR-634* (C-300961-01), a scrambled mimic (Mimic negative control #1; CN-001000-01), a miRIDIAN hairpin inhibitor for miR-634 (IH-300961-03), a scrambled miRIDIAN hairpin inhibitor (negative control; IN-001005-01) and transfection controls (miRIDIAN mimic with Dy547; CP-004500-01/miRIDIAN hairpin inhibitor with Dy547; IP-004500-01) were obtained from Dharmacon (Epsom, UK). The seeding concentrations (cells/well) in 24 well plates were for A2780 and A2780 DDP 4.5*10^4^, for OV56 and TOV21G 4*10^4^, for OAW42 3*10^4^, for TOV112D 6*10^4^ and for primary ovarian cell cultures 4*10^4^ in a final volume of 450 μL medium without antibiotics. On the day after seeding, 50 μL of a mixture of Dharmafect 1 (final concentration 0.3 % (v/v)) and mimic (final concentration 50 nM) or inhibitor (final concentration 25 nM) in serum-free medium was added dropwise to each well. Under these conditions, the transfection efficiency was over 90 % as determined using fluorescently labeled mimics. 48 h after transfection, drugs were added in varying concentrations. After 24 h of continuous drug exposure, an MTT assay was performed [52]. psiCHECK™-2 constructs were transfected using Fugene HD (Promega) according to recommendations by the manufacturer.

### FACS analysis

Forty-eight and seventy-two hours after miRNA transfection, cells were harvested and stained with FITC-Annexin V and Propidium iodide (PI) (FITC Annexin V Apoptosis Detection Kit I (BD Pharmingen, Breda, The Netherlands)) or, for cell cycle analysis, fixed in 70 % ethanol on ice, washed with PBS, then stained with PI (20 μg/mL in PBS-0.5 mL).

### Platinum measurements

Platinum measurements in cell lysates were carried out essentially as described before using Atomic Absorption Spectrophotometry (AAS) [53].

### Cytospins and immunohistochemistry

Primary ovarian cancer cell cultures were harvested using a cell scraper and collected in tubes, washed and resuspended in PBS containing 1 % BSA. 50-100 μL (depending on cell number) was used for cytospin preparation. Slides were air-dried for 30 min, fixed O/N in 10 % formalin, then stored in 70 % ethanol at 4 °C, until IHC staining. IHC staining was performed using Ventana Benchmark ULTRA automated slide stainers, and antibodies against pan keratin (Neomarkers, Fremont,CA, USA, MS-343-P), EpCAM (DAKO, Heverlee, Belgium, M0804) and p53 (DAKO, M7001).

### Pathway analysis

The entire list of predicted *miR-634* targets in Targetscan v6.0 (http://www.targetscan.org) was used for pathway analysis using the analysis wizard of DAVID Bioinformatics Resources (http://david.abcc.ncifcrf.gov/tools.jsp). Analyzed were enriched GO-terms (BP_fat; Additional file 1: Table S3) and enriched pathways (BIOCARTA, KEGG; Additional file 1: Table S4).

### Western blot analysis

Fifteen to twenty μg of total protein of each sample was subjected to SDS-PAGE/Western blotting. Specific proteins were detected with antibodies against mouse GRB2 (BD Pharmingen, 610111), mouse RSK2 (Santa Cruz, Heerhugowaard, The Netherlands, SC-9986), rabbit ERK1/2 (Cell signaling, Leiden, The Netherlands, #9102), rabbit RSK1 (Santa Cruz, SC-231), rabbit CCND1 (Thermoscientific, Etten-Leur, The Netherlands, RM-9104-S) and mouse anti-β-actin (Sigma, A5441). Secondary HRP-conjugated antibodies used were Goat-anti-mouse (Santa Cruz, SC-2005) and Goat-anti-rabbit (Jackson ImmunoResearch, Westgrove, PA, USA, 111-035-144).

### Cloning

Parts of the 3’UTR of CCND1, GRB2, ERK2, RSK1 and RSK2 (see Additional file 1: Figure S7) were PCR amplified from human genomic DNA (Promega) introducing a XhoI (5’-end) and a NotI site (3’-end). The PCR products were cloned in pCR®-Blunt, followed by XhoI and NotI restriction digests and ligation in psiCHECK™-2 (Promega). The constructs were verified by sequencing. The putative *miR-634* target sites were mutated, forming a SmaI site to efficiently screen for mutants, in the CCND1, GRB2, ERK2 and RSK2 3’UTR constructs, using the Quickchange site-directed mutagenesis kit (Stratagene; Additional file 1: Figure S7).

### Luciferase assay

Forty-eight hours after transfection with the miRNA mimics/inhibitors and the psiCHECK2 constructs, a luciferase assay (Promega; Dual-Luciferase Reporter assay) was carried out according to the manufacturer’s instructions. The *Renilla* luciferase expression was normalized on the Firefly luciferase signal.

### Statistical analysis

A paired SAM analysis [54] was performed to compare miRNA expression profiles of cisplatin sensitive and resistant ovarian (A2780/A2780 DDP), colon (HCT8/HCT8 DDP) and bladder cancer cell lines (T24/T24 DDP). Two-tailed paired sample T-tests were used to assess whether differences were consistent between the scrambled mimic and *miR-634* (Figs. 1, 2, 4d). Two way ANOVA’s were used to examine whether *miR-634* overexpression had a significant effect on drug sensitivity, independent of the ascites batch. Furthermore, the effect of *miR-634* overexpression on drug sensitivity in individual ascites cultures was assessed using paired sample T-tests (Fig. 3, Additional file 1: Figure S3 and S4). *miR-634* and CCND1, ERK2, RSK1 and RSK2 expression was examined in 535 ovarian serous cystadenoma’s (TCGA dataset, (http://cancergenome.nih.gov/). Spearman rank tests were used to correlate target gene expression in tumors with high *miR-634* expression (expression > mean + stdev) (Additional file 1: Figure S10).

## Additional file

Additional file 1: Table S1. Cisplatin sensitivity of cell lines. **Table S2.** miRNAs that show a ≥1.5 fold change of expression in cisplatin sensitive and resistant cell lines. **Table S3.** GO-terms that are enriched for miR-634 targets. **Table S4.** An overview of KEGG and Biocarta pathways that are enriched for miR-634 targets. **Figure S1.** miR-634 is the only miRNA showing consistent changes in cisplatin sensitive and resistant cell lines. **Figure S2.** Platinum levels in A2780 DDP cells transfected with miR-634 or scrambled controls. **Figure S3.** Immunohistochemical staining of primary ovarian cancer cell cultures. **Figure S4.** miR-634 enhances cisplatin sensitivity. **Figure S5.** miR-634 enhances carboplatin sensitivity. **Figure S6.** Predicted miR-634 targets. **Figure S7.** Overview of the location of the miR-634 target sites in the 3’UTR of its potential target genes. **Figure S8.** Effect of miR-634 overexpression on luciferase activity of psiCHECK™-2 reporter constructs containing part of the 3’UTR of miR-634 predicted targets. **Figure S9.** Effect of miR-634 inhibitors on luciferase activity of psiCHECK™-2 reporter constructs containing part of the 3’UTR of miR-634 predicted targets. **Figure S10.** Correlation between miR-634 expression and its target gene expression in 23 miR-634 expressing primary ovarian cancer samples (TCGA dataset). **Figure S11.** Effect of PD0325901 on cell survival and cisplatin sensitivity. **Figure S12.** Detection of miR-634, RNU43 and RNU48 by RT-PCR. (PDF 1609 kb)
