# Supplementary material for: miR-634 restores drug sensitivity in resistant ovarian cancer cells by targeting the Ras-MAPK pathway
Source: Mol Cancer. 2015 Nov 17;14:196. doi: 10.1186/s12943-015-0464-4 (PMC4650519; doi:10.1186/s12943-015-0464-4)

Supplementary legends

Supplementary table 1: Cisplatin sensitivity of cell lines

A: The IC_{50} values for cisplatin of ovarian cancer (A2780), colon cancer (HCT8) and bladder cancer (T24) cell line pairs were determined after 5 days of continuous exposure to cisplatin using an MTT assay. The IC_{50} value was estimated based on viability data of three independent experiments. Each cell line pair consists of a parental sensitive cell line and a resistant derivative (DDP). The difference in IC_{50} value between sensitive and resistant cell lines is comparable to previously published data (Goto *et al*, 1995; Kool *et al*, 1997; Kotoh *et al*,1994; Schmidt and Chaney, 1993)

B: A2780, HCT8 and T24 cells and their resistant counterparts were treated with 100 µM cisplatin for 2 hours after which total cellular platinum levels were determined using Atomic Absorption Spectrophotometry. Indicated is the average and standard deviation of three biological replicates. Note that reduced cellular platinum levels are found in the cisplatin resistant cell lines.

C: The IC_{25} values for cisplatin of ovarian cancer cell lines A2780, A2780 DDP, OAW42, OV56, TOV21G and TOV112D were determined in mock (Dharmafect 1 only) transfected cells. 48 hours after transfection, cisplatin was added and after 24 hours of continuous exposure an MTT assay was used to determine cell viability.

Supplementary table 2: miRNAs that show a ≥1.5 fold change of expression in cisplatin sensitive and resistant cell lines.

miRNA expression was compared between sensitive and resistant ovarian cancer (A2780/A2780 DDP), colon cancer (HCT8/HCT8 DDP) and bladder cancer (T24/T24 DDP). Listed are all miRNAs that show a fold change ≥1.5 in the resistant cell line relative to its sensitive counterpart, as indicated by a red (expression is higher in the resistant cell line) or a green color (expression is lower in the resistant cell line).

Supplementary table 3: GO-terms that are enriched for *miR-634* targets

Supplementary table 4: An overview of KEGG and Biocarta pathways that are enriched for *miR-634* targets.

Supplementary figure 1: *miR-634* is the only miRNA showing consistent changes in cisplatin sensitive and resistant cell lines. miRNA expression was compared between sensitive and resistant ovarian cancer (A2780/A2780 DDP), colon cancer (HCT8/HCT8 DDP) and bladder cancer (T24/T24 DDP) using pairwise SAM analysis (FDR=0.000).

Supplementary figure 2: Platinum levels in A2780 DDP cells transfected with miR-634 or scrambled controls. To investigate whether modulation of miR-634 levels affect the cellular uptake of cisplatin in ovarian cancer cells A2780 DDP cells were transfected with scrambled miRNA (mneg) or miR-634 mimic (m634). 48 hours after transfection, cells were treated with 80 µM, 125 µM cisplatin or no cisplatin. Cells were washed and harvested at 2H, 12H and 24H. Four biological replicates were pooled before protein content was measured and platinum levels were determined using Atomic Absorption Spectrophotometry. After 2 hours of cisplatin exposure no difference in intracellular platinum levels was observed between *miR-634* overexpressing A2780 DDP cells and control transfectants. Furthermore, at 6H and 12H of cisplatin exposure no consistent difference in platinum content between the *miR-634* and control transfected cells was detected.

Supplementary figure 3: Immunohistochemical staining of primary ovarian cancer cell cultures. Ovarian tumor cells were cultured from ascites fluid obtained from chemoresistant patients and stained for the epithelial markers EpCAM (aka BerEp4) and pan-Keratin, and the tumor-specific marker p53. The bar represents 100 µm.

Supplementary figure 4: *miR-634* enhances cisplatin sensitivity. Depicted are the results for all ascites derived primary ovarian cell cultures. For each culture, a representative experiment is presented (A-B: culture 1-2, n=1; C-G: culture 3-7, n=3). * = $p<0.05$, ** = $p < 0.01$.

Supplementary figure 5: *miR-634* enhances carboplatin sensitivity. Depicted are the results of a representative experiment. Culture 3 (A): n=3, culture 4 (B): n=1, culture 5 (C): n=1, culture 7 (D): n=3.

Supplementary figure 6: Predicted *miR-634* targets

A: *MiR-634* is predicted to target several components of the Insulin signaling pathway (pink), the mTOR signaling pathway (blue) and the Ras-MAPK pathway (yellow). Predicted targets, INSR, IRS, PI3K, RICTOR, EIF4E, GRB2, N-RAS, RAF1, ERK2, RSK1 and RSK2 are marked by a star.

B: An overview of the 10 GO terms that are most enriched for putative *miR-634* targets. The red colors depict GO terms related to cell cycle regulation, the yellow colors GO terms related to regulation of proliferation. All other bars are blue.

Supplementary figure 7: Overview of the location of the *miR-634* target sites in the 3'UTR of its potential target genes.

A: Overview of the location of the cloned regions and the miR-634 target sites within the 3´UTR of CCND1, GRB2, ERK2, RSK1 and RSK2

B: Overview of the miR-634 target sites and primers used to clone the 3´UTR and to mutate the miR-634 target site

Supplementary figure 8: Effect of *miR-634* overexpression on luciferase activity of psiCHECK™-2 reporter constructs containing part of the 3'UTR of *miR-634* predicted targets. A2780 DDP cells were transfected with a scrambled mimic or a *miR-634* mimic.

After 8 hours, the same cells were transfected with *Renilla* luciferase reporter constructs (psiCHECK™-2) containing 3'UTR regions of 500 bp surrounding the predicted miR-634 target sites of various target genes (Supplementary figure 7). *ERK2* and *RSK2* contain respectively three or two *miR-634* binding sites, and for each site the 3'UTR region surrounding the target site was cloned. The *Renilla* luciferase activity was measured and normalized using the Firefly luciferase activity. The relative luciferase activity of cells transfected with the empty vector (EV) was set at 1. Depicted are the average values ± SD (n= 3).

Supplementary figure 9: Effect of *miR-634* inhibitors on luciferase activity of psiCHECK™-2 reporter constructs containing part of the 3'UTR of *miR-634* predicted targets. A2780 DDP cells were transfected with a scrambled inhibitor or a *miR-634* antisense inhibitor. After 8 hours, the same cells were transfected with *Renilla* luciferase constructs (psiCHECK™-2) containing 3'UTR regions of 500 bp surrounding the predicted *miR-634* target sites of various target genes (Supplementary figure 7). The *Renilla* luciferase activity was measured and normalized using the Firefly luciferase activity. The relative luciferase activity of cells transfected with the scrambled inhibitor was set at 1. Depicted are the average values ± SD (n= 4).

Supplementary figure 10: Correlation between *miR-634* expression and its target gene expression in 23 *miR-634* expressing primary ovarian cancer samples (TCGA dataset). Spearman rank correlation tests were performed to assess the relation between *miR-634* expression (miRNA dataset) and target gene expression (mRNA dataset).

Supplementary figure 11: Effect of PD0325901 on cell survival and cisplatin sensitivity
A: Treatment with the MEK inhibitor PD0325901 (1 µM) decreases cell viability 48 H after treatment. Depicted is the average viability (n=2). Error bars represents the standard deviation.

B: Treatment with the MEK inhibitor PD0325901 augments cisplatin sensitivity. Cells were cotreated with the indicated amounts of cisplatin and 1 µM PD0325901. After 24 H the cell viability was determined. Depicted is the average viability (n=2). Error bars represent the standard deviation.

Supplementary figure 12: Detection of miR-634, RNU43 and RNU48 by RT-PCR

A: Detection of miR-634 by Taqman assay (ID # 001576; Applied Biosystems). No expression of miR-634 could be detected by RT-PCR in A2780 and A2780-ddp. In A2780-ddp transfected with a miR-634 mimic (A2780-ddp M634, n=3), miR-634 was readily detectable.

B: Detection of RNU43 and RNU 48 by Taqman assay (ID # 001095 and ID # 001006, respectively). Both RNU43 and RNU48 expression was detected at the expected levels in both A2780, A2780-ddp and A2780-ddp transfected with a miR-634 mimic.

References:

Goto, S., Yoshida, K., Morikawa, T., Urata, Y., Suzuki, K., Kondo, T., 1995. Augmentation of transport for cisplatin-glutathione adduct in cisplatin-resistant cancer cells. Cancer Res 55, 4297-4301.

Kool, M., de Haas, M., Scheffer, G.L., Scheper, R.J., van Eijk, M.J., Juijn, J.A., Baas, F., Borst, P., 1997. Analysis of expression of cMOAT (MRP2), MRP3, MRP4, and MRP5, homologues of the multidrug resistance-associated protein gene (MRP1), in human cancer cell lines. Cancer Res 57, 3537-3547.

Kotoh, S., Naito, S., Yokomizo, A., Kumazawa, J., Asakuno, K., Kohno, K., Kuwano, M., 1994. Increased expression of DNA topoisomerase I gene and collateral sensitivity to camptothecin in human cisplatin-resistant bladder cancer cells. Cancer Res 54, 3248-3252.

Schmidt, W., Chaney, S.G., 1993. Role of carrier ligand in platinum resistance of human carcinoma cell lines. Cancer Res 53, 799-805.

Supplementary table 1

A

MTT assay (5 day cisplatin exposure)

Cell lines	IC_{50} (μM)	Fold resistance
A2780	2.4	
A2780 DDP	17.9	7.5
HCT8	6.8	
HCT8 DDP	19.5	2.9
T24	1.4	
T24 DDP 10	12.0	8.6

B

Cisplatin levels (2 hour 100 μM cisplatin)

Cell lines	ng Pt/ μg Protein
A2780	73.9 ± 4.4
A2780 DDP	41.8 ± 15.7
HCT8	52.6 ± 14.6
HCT8 DDP	30.2 ± 4.56
T24	47.0 ± 7.5
T24 DDP 10	38.3 ± 2.9

C

MTT assay (24 hour exposure)

Cell lines	IC_{25} (μM)
A2780	10
A2780 DDP	80
OAW42	20
OV56	20
TOV21G	50
TOV112D	5

Supplementary table 2

Annotation	HCT8	T24	A2780
hsa-miR-634	-1.5	-2.8	-1.5
hsa-miR-214	-1.3	-1.6	-2.1
hsa-miR-10b	1.2	-1.5	-2.9
hsa-miR-493-star	1	-1.5	-2.7
hsa-miR-382	1.3	-2.2	-1.5
hsa-miR-23a	1	-1.8	-1.5
hsa-let-7d	1.2	-1.5	-1.6
hsa-miR-19b	-1.8	-1.1	-3.3
hsa-miR-668	-1.5	-1.2	-1.5
hsa-miR-18b	-1.6	1.1	-4.4
hsa-miR-106a	-1.9	1.2	-3.9
hsa-miR-17	-1.7	1	-3.6
hsa-miR-19a	-2.1	1	-2.5
hsa-miR-186-star	-1.7	2.1	-2.4
hsa-miR-363-star	-1.5	1.6	-1.7
hsa-miR-510	-1.5	-1.5	1.1
hsa-miR-16	-2.1	1.3	-1.1
hsa-miR-130b	-2.0	1.1	1.1
hsa-miR-425-star	-1.9	-1.4	1.1
hsa-miR-26a	-1.9	1.2	1.1
hsa-miR-627	-1.9	1.3	1.3
hsa-miR-320	-1.8	-1.1	1.4
hsa-miR-10a-star	-1.8	1	1.1
hsa-miR-17-star	-1.7	-1.3	1.1
hsa-miR-183-star	-1.7	-1.2	1.1
hsa-miR-329	-1.7	1.1	-1.1
hsa-miR-801	-1.6	1	1.3
hsa-let-7c-star	-1.5	1.2	1.1
hsa-miR-30c	-1.5	1.3	1
hsa-miR-200b	-1.5	1.3	1.1
hsa-miR-181a-2-star	-1.5	1.3	1.3
hsa-miR-193a-3p	-1.5	1.4	1.3
hsa-miR-516a-5p	-1.1	-3	-1.1
hsa-miR-302c-star	1	-2.8	1
hsa-miR-194-star	-1.2	-2.6	1.3
hsa-miR-493	1	-2.4	-1.1
hsa-miR-338-3p	1.1	-2.3	1.3
hsa-miR-501-5p	1.2	-1.9	1.2
hsa-miR-675	-1.2	-1.8	1.3
hsa-miR-516b	-1.1	-1.8	1.4
hsa-miR-34c-5p	-1.1	-1.7	-1.1
hsa-miR-485-5p	1	-1.6	-1.3
hsa-miR-520d-5p	1.1	-1.6	-1.1
hsa-miR-24-1-star	1	-1.6	1
hsa-miR-520a-5p	1.1	-1.6	1
hsa-miR-379	1.2	-1.6	1.1
hsa-miR-23b	1.1	-1.6	1.4
hsa-miR-452	-1.2	-1.5	1
hsa-let-7b	-1.1	-1.5	1
hsa-miR-10a	1	-1.5	-1.1
hsa-miR-342-5p	-1.3	-1.5	1.2
hsa-miR-184	-1.2	-1.5	1.2
hsa-miR-155	-1.1	-1.5	1.3
hsa-miR-361-3p	1.3	-1.5	-1.1
hsa-miR-532-3p	1	-1.5	1.2
hsa-let-7b-star	1.3	-1.5	1
hsa-miR-125b-2-star	1.2	-1.5	1.1
hsa-miR-371-5p	1	-1.5	1.4
hsa-miR-199a-5p	1	1.2	-36.7
hsa-miR-199a-3p; hsa-miR-199b-3p	1.3	1.4	-18.3
hsa-miR-942	-1.2	-1.1	-12.3
hsa-miR-335	-1.3	1.2	-7.7
hsa-miR-20b	1.1	1	-4.2
hsa-miR-412	1	1.3	-4
hsa-miR-645	-1.1	1	-3.3
hsa-miR-196a	-1.2	1	-2.7
hsa-miR-135b-star	-1.2	1.1	-2
hsa-miR-130a	-1.2	1.3	-2
hsa-miR-338-5p	1	1.2	-2
hsa-miR-29b	1	-1.3	-1.9
hsa-miR-18a	-1.2	-1.4	-1.7
hsa-miR-20a	-1.4	1	-1.7
hsa-miR-593-star	1.1	-1.1	-1.7
hsa-miR-339-5p	1	1.1	-1.7
hsa-miR-297	1.4	1	-1.7
hsa-miR-105	1	-1.4	-1.5
hsa-miR-30c-2-star	1.2	-1.4	-1.5
hsa-miR-141-star	-1.3	1.4	-1.5
hsa-miR-513a-5p	1.2	-1.1	-1.5
hsa-miR-191	-1.2	1.4	-1.5
hsa-miR-886-5p	-1.1	1.3	-1.5
hsa-miR-647	1.3	1.1	-1.5
hsa-miR-542-3p	-1.2	2	-1.6
hsa-miR-300	-1.3	2.5	-1.6
hsa-miR-421	1.1	1.9	-2.3
hsa-miR-92a-2-star	2.1	1.2	-1.5
hsa-miR-449a	1.6	-1.4	-1.6
hsa-miR-522	1.6	-1.6	-1.2
hsa-miR-22	-2.3	1.6	1.1

Annotation	HCT8	T24	A2780
hsa-miR-628-3p	-1.8	1.6	1
hsa-miR-31	-1.7	2.2	1
hsa-miR-340-star	-1.5	2.7	-1.1
hsa-miR-378	-1.5	1.6	1
hsa-miR-636	-1.5	1.5	1.1
hsa-miR-886-3p	-1.5	1.9	1
hsa-miR-141	-1.9	1.1	7.8
hsa-miR-518e	-1.7	-1.2	2.2
hsa-miR-497	-1.6	1.1	1.6
hsa-miR-190b	-1.5	-1.2	1.5
hsa-miR-923	-35.1	1.7	1.7
hsa-miR-215	2.3	2	-8.1
hsa-miR-433	1.7	1.8	1
hsa-miR-518a-3p	1.5	-1.3	1.1
hsa-miR-197	1.5	-1.2	-1.1
hsa-miR-455-3p	1.5	-1.2	-1.1
hsa-miR-330-5p	1.5	-1.2	1
hsa-miR-34a	1.5	-1.1	1
hsa-miR-220b	1.5	1	1
hsa-miR-766	1.5	1	1
hsa-miR-622	1.5	1.2	1.2
hsa-miR-576-5p	1.6	-1.3	-1.3
hsa-miR-374a-star	1.6	-1.1	-1.1
hsa-miR-921	1.6	1	1.2
hsa-miR-625-star	1.8	-1.3	-1.1
hsa-miR-148b-star	2	-1.2	-1.1
hsa-miR-708-star	2	1	1.2
hsa-miR-16-2-star	2.1	1.1	1
hsa-miR-708	-1.3	1.5	1.1
hsa-miR-151-3p	-1.1	1.5	1.1
hsa-miR-143	1.1	1.5	1.1
hsa-miR-324-3p	1.1	1.5	1.1
hsa-miR-637	-1.4	1.5	1.2
hsa-miR-125a-5p	-1.2	1.5	1.2
hsa-miR-935	1.1	1.5	1.2
hsa-miR-328	1.2	1.5	1.2
hsa-miR-485-3p	-1.2	1.6	-1.1
hsa-miR-181b	1.2	1.6	1
hsa-miR-193b	-1.1	1.6	1.1
hsa-miR-124-star	1.3	1.6	1.3
hsa-miR-18b-star	1	1.6	1.4
hsa-miR-29a	-1.2	1.7	-1.3
hsa-miR-21	1.1	1.7	-1.3
hsa-miR-885-5p	1.2	1.7	-1.1
hsa-miR-601	1.1	1.7	1.1
hsa-miR-130b-star	1.1	1.7	1.3
hsa-miR-1	-1.4	1.8	1.1
hsa-miR-659	1.2	1.8	1.3
hsa-miR-146a	-1.1	1.8	1.4
hsa-miR-576-3p	-1.2	1.9	1.1
hsa-miR-340	1.1	2	1.1
hsa-miR-580	1.1	2	1.1
hsa-miR-21-star	1.2	2	1.1
hsa-miR-518b	1.2	2.1	1.4
hsa-miR-488	-1.1	2.2	1.2
hsa-miR-626	-1.3	2.4	1.3
hsa-miR-135a	1.2	2.5	-1.1
hsa-miR-502-5p	1.1	-1.4	1.6
hsa-miR-551b-star	1	-1.2	1.6
hsa-miR-885-3p	1.2	-1.2	1.7
hsa-miR-210	-1.1	-1.2	1.9
hsa-miR-219-2-3p	1.1	-1.1	1.5
hsa-miR-575	1.1	-1.1	1.5
hsa-miR-93	-1.2	-1.1	1.6
hsa-miR-106b-star	-1.1	-1.1	1.6
hsa-miR-122-star	-1.1	-1.1	1.6
hsa-miR-608	1.1	-1.1	1.7
hsa-miR-610	1	1	1.5
hsa-miR-632	1.2	1	1.5
hsa-miR-377-star	1.1	1	1.6
hsa-miR-132-star	1.2	1	1.6
hsa-miR-526b-star	-1.3	1.1	1.5
hsa-miR-491-5p	-1.1	1.1	1.5
hsa-miR-550-star	-1.1	1.1	1.9
hsa-miR-499-3p	-1.4	1.2	1.5
hsa-miR-220c	-1.1	1.2	1.5
hsa-miR-200a	-1.3	1.2	1.6
hsa-miR-615-5p	1.3	1.2	1.7
hsa-miR-936	1.4	1.2	1.9
hsa-miR-222	1	1.4	2
hsa-miR-365	1.2	1.4	2
hsa-miR-631	-1.1	1.4	2.1
hsa-miR-200c	-1.4	1.4	2.6
hsa-miR-424-star	1.6	-1.2	1.5
hsa-miR-29b-2-star	1.6	1.4	1.5
hsa-miR-106b	-1.4	1.6	1.6
hsa-miR-182	-1.2	1.9	1.6
hsa-miR-203	-1.3	1.9	1.7
hsa-miR-144-star	-1.3	3	2.2

Supplementary table 3

Category	Term	Count	%	PValue	Putative miR-634 targets	List Total	Pop Hits	Pop Total	Fold Enric	Bonferroni	Benjamini	FDR
GOTERM_BP_FAT	GO:0045737~positive regulation of cyclin-dependent protein kinase activity	5	1.7301	3.76E-06	NM_053056, NM_002709, NM_001759, NM_003183, NM_002648	211	8	13528	40.0711	0.00624	0.00624	0.00633
GOTERM_BP_FAT	GO:0008284~positive regulation of cell proliferation	21	7.26644	7.02E-06	NM_000208, NM_020338, NM_053056, NM_005584, NM_005544, NM_001042599, NM_001759, NM_030661, NM_003183, NM_002745, NM_001001342, NM_001102426, NM_003873, NM_032682, NM_001024736, NM_001114979, NM_002709, NM_001033910, NM_001130916, NM_032109, NM_002524	211	414	13528	3.25215	0.01162	0.00583	0.01181
GOTERM_BP_FAT	GO:0051347~positive regulation of transferase activity	14	4.84429	9.62E-05	NM_000208, NM_053056, NM_005544, NM_001759, NM_003183, NM_002648, NM_022766, NM_020755, NM_002709, NM_005921, NM_001130916, NM_006609, NM_001079874, NM_002730	211	240	13528	3.73997	0.14805	0.05201	0.16179
GOTERM_BP_FAT	GO:0007507~heart development	13	4.49827	1.35E-04	NM_002957, NM_004423, NM_000208, NM_020338, NM_001042599, NM_000264, NM_000801, NM_004555, NM_001114107, NM_003873, NM_032682, NM_001130916, NM_001105077	211	215	13528	3.87664	0.20177	0.05478	0.22747
GOTERM_BP_FAT	GO:0019220~regulation of phosphate metabolic process	20	6.92042	2.02E-04	NM_000208, NM_053056, NM_005544, NM_000333, NM_001759, NM_000801, NM_003183, NM_002648, NM_022766, NM_152756, NM_006761, NM_018724, NM_002709, NM_012479, NM_005921, NM_001130916, NM_000859, NM_006609, NM_001079874, NM_002730	211	485	13528	2.64387	0.28592	0.06513	0.33972
GOTERM_BP_FAT	GO:0051174~regulation of phosphorus metabolic process	20	6.92042	2.02E-04	NM_000208, NM_053056, NM_005544, NM_000333, NM_001759, NM_000801, NM_003183, NM_002648, NM_022766, NM_152756, NM_006761, NM_018724, NM_002709, NM_012479, NM_005921, NM_001130916, NM_000859, NM_006609, NM_001079874, NM_002730	211	485	13528	2.64387	0.28592	0.06513	0.33972
GOTERM_BP_FAT	GO:0042127~regulation of cell proliferation	27	9.34256	2.18E-04	NM_000208, NM_005544, NM_005584, NM_053056, NM_001042599, NM_001759, NM_003183, NM_000264, NM_001102426, NM_003873, NM_032682, NM_001114979, NM_002709, NM_001033910, NM_001130916, NM_004779, NM_001105077, NM_002524, NM_002957, NM_020338, NM_030661, NM_002745, NM_001001342, NM_000214, NM_001024736, NM_002359, NM_032109	211	787	13528	2.19958	0.30433	0.05869	0.36602
GOTERM_BP_FAT	GO:0000082~G1/S transition of mitotic cell cycle	7	2.42215	2.22E-04	NM_053056, NM_002709, NM_001759, NM_003183, NM_002648, NM_015981, NM_004302	211	56	13528	8.01422	0.30852	0.05134	0.3721
GOTERM_BP_FAT	GO:0033674~positive regulation of kinase activity	13	4.49827	2.64E-04	NM_000208, NM_053056, NM_005544, NM_001759, NM_003183, NM_022766, NM_002648, NM_002709, NM_005921, NM_001130916, NM_006609, NM_001079874, NM_002730	211	231	13528	3.60813	0.35574	0.05347	0.44329
GOTERM_BP_FAT	GO:0046777~protein amino acid autophosphorylation	8	2.76817	3.48E-04	NM_000208, NM_001396, NM_002648, NM_005921, NM_015981, NM_001130916, NM_002730, NM_004302	211	85	13528	6.03423	0.44005	0.0624	0.58429
GOTERM_BP_FAT	GO:0042325~regulation of phosphorylation	19	6.57439	3.52E-04	NM_000208, NM_053056, NM_005544, NM_000333, NM_001759, NM_003183, NM_000801, NM_002648, NM_022766, NM_152756, NM_018724, NM_002709, NM_012479, NM_005921, NM_001130916, NM_000859, NM_006609, NM_001079874, NM_002730	211	466	13528	2.61408	0.44361	0.05694	0.5907
GOTERM_BP_FAT	GO:0032268~regulation of cellular protein metabolic process	19	6.57439	4.36E-04	NM_000208, NM_001130678, NM_053056, NM_001079533, NM_001759, NM_001161576, NM_003183, NM_000801, NM_000624, NM_002745, NM_006775, NM_152756, NM_145808, NM_006761, NM_001134649, NM_018724, NM_005921, NM_001130916, NM_006609	211	474	13528	2.56996	0.51609	0.06386	0.7308
GOTERM_BP_FAT	GO:0051338~regulation of transferase activity	16	5.53633	7.12E-04	NM_000208, NM_053056, NM_005544, NM_001759, NM_003183, NM_002648, NM_022766, NM_020755, NM_002709, NM_012479, NM_005921, NM_001130916, NM_000859, NM_006609, NM_001079874, NM_002730	211	372	13528	2.75758	0.6946	0.09412	1.19144
GOTERM_BP_FAT	GO:0010604~positive regulation of macromolecule metabolic process	27	9.34256	7.95E-04	NM_000208, NM_005544, NM_053056, NM_003206, NM_001759, NM_000801, NM_003183, NM_004555, NM_152756, NM_001114979, NM_018724, NM_001130916, NM_001079846, NM_001105077, NM_002957, NM_004423, NM_020338, NM_000333, NM_001161576, NM_002745, NM_001001342, NM_001083962, NM_001024736, NM_001126054, NM_001142418, NM_005921, NM_001098833	211	857	13528	2.01992	0.73389	0.09682	1.32886
GOTERM_BP_FAT	GO:0044093~positive regulation of molecular function	21	7.26644	8.06E-04	NM_000208, NM_053056, NM_005544, NM_001008224, NM_001759, NM_001142339, NM_000801, NM_003183, NM_002648, NM_022766, NM_000684, NM_015981, NM_001743, NM_002564, NM_020755, NM_002709, NM_005921, NM_001130916, NM_006609, NM_001079874, NM_002730	211	586	13528	2.29759	0.73873	0.09142	1.34716
GOTERM_BP_FAT	GO:0043549~regulation of kinase activity	15	5.19031	0.00139	NM_000208, NM_053056, NM_005544, NM_001759, NM_003183, NM_002648, NM_022766, NM_002709, NM_012479, NM_005921, NM_001130916, NM_000859, NM_006609, NM_001079874, NM_002730	211	357	13528	2.69385	0.90062	0.14266	2.30606
GOTERM_BP_FAT	GO:0001932~regulation of protein amino acid phosphorylation	10	3.46021	0.00152	NM_000208, NM_053056, NM_018724, NM_001759, NM_003183, NM_000801, NM_005921, NM_001130916, NM_006609, NM_152756	211	173	13528	3.706	0.91991	0.14597	2.51892
GOTERM_BP_FAT	GO:0045787~positive regulation of cell cycle	6	2.07612	0.00187	NM_000208, NM_053056, NM_002709, NM_001759, NM_003183, NM_002648	211	57	13528	6.74882	0.9556	0.16741	3.09836
GOTERM_BP_FAT	GO:0010647~positive regulation of cell communication	14	4.84429	0.00191	NM_000208, NM_022130, NM_005544, NM_001042599, NM_003183, NM_000801, NM_152756, NM_000214, NM_001114979, NM_018724, NM_001033910, NM_001130916, NM_002524, NM_004302	211	329	13528	2.72824	0.95841	0.16194	3.16236
GOTERM_BP_FAT	GO:0051056~regulation of small GTPase mediated signal transduction	12	4.15225	0.00195	NM_152545, NM_015310, NM_001146213, NM_014247, NM_001144856, NM_152756, NM_012180, NM_001102426, NM_002524, NM_144628, NM_001079874, NM_020340	211	252	13528	3.05304	0.96147	0.1575	3.23692
GOTERM_BP_FAT	GO:0009967~positive regulation of signal transduction	13	4.49827	0.00223	NM_000208, NM_022130, NM_005544, NM_003183, NM_000801, NM_152756, NM_000214, NM_001114979, NM_018724, NM_001033910, NM_001130916, NM_002524, NM_004302	211	295	13528	2.82535	0.97572	0.16965	3.68749
GOTERM_BP_FAT	GO:0045860~positive regulation of protein kinase activity	11	3.80623	0.00255	NM_000208, NM_053056, NM_002709, NM_001759, NM_003183, NM_002648, NM_022766, NM_005921, NM_001130916, NM_006609, NM_002730	211	223	13528	3.16256	0.98567	0.18304	4.19942
GOTERM_BP_FAT	GO:0001934~positive regulation of protein amino acid phosphorylation	7	2.42215	0.0026	NM_000208, NM_053056, NM_018724, NM_001759, NM_003183, NM_001130916, NM_152756	211	89	13528	5.04265	0.98685	0.17871	4.28239
GOTERM_BP_FAT	GO:0010557~positive regulation of macromolecule biosynthetic process	21	7.26644	0.00296	NM_000208, NM_002957, NM_004423, NM_020338, NM_003206, NM_005544, NM_000333, NM_001161576, NM_002745, NM_004555, NM_001001342, NM_001083962, NM_001024736, NM_001114979, NM_001142418, NM_001126054, NM_005921, NM_001130916, NM_001098833, NM_001079846, NM_001105077	211	654	13528	2.0587	0.99282	0.19315	4.86573
GOTERM_BP_FAT	GO:0043085~positive regulation of catalytic activity	18	6.22837	0.0031	NM_000208, NM_053056, NM_005544, NM_001008224, NM_001759, NM_001142339, NM_003183, NM_002648, NM_022766, NM_000684, NM_002564, NM_020755, NM_002709, NM_005921, NM_001130916, NM_006609, NM_001079874, NM_002730	211	520	13528	2.21932	0.99428	0.19361	5.08488
GOTERM_BP_FAT	GO:0051270~regulation of cell motion	10	3.46021	0.00315	NM_000208, NM_005544, NM_001042599, NM_003183, NM_001087, NM_005921, NM_001130916, NM_002745, NM_000214, NM_003873	211	193	13528	3.32196	0.99477	0.1895	5.1693
GOTERM_BP_FAT	GO:0032535~regulation of cellular component size	12	4.15225	0.00344	NM_001114979, NM_005720, NM_001130487, NM_006779, NM_003183, NM_005921, NM_005717, NM_001130916, NM_145808, NM_152756, NM_003873, NM_004302	211	271	13528	2.83898	0.99676	0.19781	5.62652
GOTERM_BP_FAT	GO:0007242~intracellular signaling cascade	33	11.4187	0.00351	NM_000208, NM_053056, NM_005544, NM_004161, NM_001142339, NM_033026, NM_006761, NM_152545, NM_001114979, NM_002564, NM_182663, NM_005737, NM_004586, NM_001130916, NM_198686, NM_002524, NM_002957, NM_004423, NM_016248, NM_003010, NM_001008224, NM_018110, NM_002649, NM_000684, NM_002745, NM_002086, NM_014247, NM_001160226, NM_005921, NM_006609, NM_001079874, NM_002730, NM_012425	211	1256	13528	1.68452	0.99712	0.19476	5.73876
GOTERM_BP_FAT	GO:0006468~protein amino acid phosphorylation	21	7.26644	0.0037	NM_000208, NM_001258, NM_001396, NM_053056, NM_001042599, NM_003010, NM_002648, NM_015981, NM_002649, NM_002745, NM_003913, NM_001126054, NM_020791, NM_080836, NM_004586, NM_005921, NM_001130916, NM_006609, NM_152835, NM_002730, NM_004302	211	667	13528	2.01857	0.9979	0.19762	6.03954
GOTERM_BP_FAT	GO:0042327~positive regulation of phosphorylation	7	2.42215	0.00399	NM_000208, NM_053056, NM_018724, NM_001759, NM_003183, NM_001130916, NM_152756	211	97	13528	4.62677	0.99871	0.20509	6.50498
GOTERM_BP_FAT	GO:0051272~positive regulation of cell motion	7	2.42215	0.0042	NM_000208, NM_005544, NM_001042599, NM_003183, NM_001087, NM_001130916, NM_002745	211	98	13528	4.57955	0.99909	0.20815	6.83053
GOTERM_BP_FAT	GO:0010562~positive regulation of phosphorus metabolic process	7	2.42215	0.00463	NM_000208, NM_053056, NM_018724, NM_001759, NM_003183, NM_001130916, NM_152756	211	100	13528	4.48796	0.99956	0.22073	7.51528
GOTERM_BP_FAT	GO:0045937~positive regulation of phosphate metabolic process	7	2.42215	0.00463	NM_000208, NM_053056, NM_018724, NM_001759, NM_003183, NM_001130916, NM_152756	211	100	13528	4.48796	0.99956	0.22073	7.51528
GOTERM_BP_FAT	GO:0031328~positive regulation of cellular biosynthetic process	21	7.26644	0.00496	NM_000208, NM_002957, NM_004423, NM_020338, NM_003206, NM_005544, NM_000333, NM_001161576, NM_002745, NM_004555, NM_001001342, NM_001083962, NM_001024736, NM_001114979, NM_001142418, NM_001126054, NM_005921, NM_001130916, NM_001098833, NM_001079846, NM_001105077	211	685	13528	1.96553	0.99975	0.228	8.02725
GOTERM_BP_FAT	GO:0048732~gland development	8	2.76817	0.0051	NM_001114979, NM_003206, NM_005544, NM_001042599, NM_030661, NM_000264, NM_001130916, NM_032109	211	135	13528	3.79933	0.9998	0.22751	8.24783
GOTERM_BP_FAT	GO:0015807~L-amino acid transport	4	1.38408	0.00519	NM_002111, NM_020755, NM_012244, NM_001031681	211	23	13528	11.1502	0.99983	0.22508	8.38808
GOTERM_BP_FAT	GO:0051329~interphase of mitotic cell cycle	7	2.42215	0.00535	NM_053056, NM_002709, NM_001759, NM_003183, NM_002648, NM_015981, NM_004302	211	103	13528	4.35724	0.99987	0.22519	8.62863
GOTERM_BP_FAT	GO:0046578~regulation of Ras protein signal transduction	10	3.46021	0.00551	NM_015310, NM_001146213, NM_001144856, NM_152756, NM_012180, NM_001102426, NM_002524, NM_144628, NM_001079874, NM_020340	211	210	13528	3.05304	0.9999	0.2256	8.88139
GOTERM_BP_FAT	GO:0009891~positive regulation of biosynthetic process	21	7.26644	0.00581	NM_000208, NM_002957, NM_004423, NM_020338, NM_003206, NM_005544, NM_000333, NM_001161576, NM_002745, NM_004555, NM_001001342, NM_001083962, NM_001024736, NM_001114979, NM_001142418, NM_001126054, NM_005921, NM_001130916, NM_001098833, NM_001079846, NM_001105077	211	695	13528	1.93725	0.99994	0.23054	9.3338
GOTERM_BP_FAT	GO:0043627~response to estrogen stimulus	7	2.42215	0.00587	NM_000208, NM_053056, NM_001759, NM_000264, NM_001130916, NM_002745, NM_001014797	211	105	13528	4.27425	0.99994	0.22735	9.42983
GOTERM_BP_FAT	GO:0051325~interphase	7	2.42215	0.00614	NM_053056, NM_002709, NM_001759, NM_003183, NM_002648, NM_015981, NM_004302	211	106	13528	4.23393	0.99996	0.23132	9.84844
GOTERM_BP_FAT	GO:0030857~negative regulation of epithelial cell differentiation	3	1.03806	0.00631	NM_001114979, NM_053056, NM_001130916	211	8	13528	24.0427	0.99997	0.23177	10.1092
GOTERM_BP_FAT	GO:0048729~tissue morphogenesis	9	3.11419	0.00704	NM_002957, NM_001114979, NM_003206, NM_002111, NM_000801, NM_000264, NM_001166283, NM_000214, NM_002730	211	180	13528	3.20569	0.99999	0.24934	11.2039
GOTERM_BP_FAT	GO:0007568~aging	7	2.42215	0.00733	NM_002957, NM_001114979, NM_020338, NM_002111, NM_001130916, NM_002086, NM_002524	211	110	13528	4.07997	1	0.25302	11.645
GOTERM_BP_FAT	GO:0045859~regulation of protein kinase activity	13	4.49827	0.0077	NM_000208, NM_053056, NM_001759, NM_003183, NM_022766, NM_002648, NM_002709, NM_012479, NM_005921, NM_001130916, NM_000859, NM_006609, NM_002730	211	345	13528	2.41588	1	0.2588	12.2019
GOTERM_BP_FAT	GO:0000165~MAPKKK cascade	9	3.11419	0.008	NM_000208, NM_014247, NM_003010, NM_018110, NM_005921, NM_001130916, NM_002745, NM_006609, NM_002086	211	184	13528	3.136	1	0.26206	12.6388
GOTERM_BP_FAT	GO:0030833~regulation of actin filament polymerization	5	1.7301	0.00974	NM_005720, NM_006779, NM_005921, NM_005717, NM_152756	211	54	13528	5.93646	1	0.30384	15.1843
GOTERM_BP_FAT	GO:0000079~regulation of cyclin-dependent protein kinase activity	5	1.7301	0.00974	NM_053056, NM_002709, NM_001759, NM_003183, NM_002648	211	54	13528	5.93646	1	0.30384	15.1843
GOTERM_BP_FAT	GO:0030856~regulation of epithelial cell differentiation	4	1.38408	0.01001	NM_001114979, NM_053056, NM_018724, NM_001130916	211	29	13528	8.84328	1	0.30521	15.5716
GOTERM_BP_FAT	GO:0048545~response to steroid hormone stimulus	9	3.11419	0.0102	NM_000208, NM_002957, NM_053056, NM_001042599, NM_001759, NM_000264, NM_001130916, NM_002745, NM_001014797	211	192	13528	3.00533	1	0.30443	15.837
GOTERM_BP_FAT	GO:0032270~positive regulation of cellular protein metabolic process	10	3.46021	0.01064	NM_000208, NM_053056, NM_018724, NM_001759, NM_001161576, NM_003183, NM_000801, NM_001130916, NM_002745, NM_152756	211	233	13528	2.75166	1	0.30988	16.4642
GOTERM_BP_FAT	GO:0009719~response to endogenous stimulus	14	4.84429	0.01069	NM_002957, NM_000208, NM_053056, NM_005544, NM_001042599, NM_001142339, NM_001759, NM_003183, NM_000264, NM_002745, NM_002086, NM_001014797, NM_001130916, NM_002730	211	405	13528	2.21628	1	0.30599	16.5452
GOTERM_BP_FAT	GO:0043467~regulation of generation of precursor metabolites and energy	4	1.38408	0.011	NM_000208, NM_005544, NM_002709, NM_002730	211	30	13528	8.5485	1	0.30804	16.9767
GOTERM_BP_FAT	GO:0014070~response to organic cyclic substance	7	2.42215	0.01145	NM_002957, NM_053056, NM_001759, NM_001142339, NM_000801, NM_000264, NM_001130916	211	121	13528	3.70906	1	0.31328	17.6087
GOTERM_BP_FAT	GO:0030335~positive regulation of cell migration	6	2.07612	0.01245	NM_000208, NM_005544, NM_001042599, NM_003183, NM_001087, NM_002745	211	89	13528	4.32227	1	0.33046	19.0063
GOTERM_BP_FAT	GO:0007243~protein kinase cascade	13	4.49827	0.0129	NM_000208, NM_005544, NM_016248, NM_003010, NM_018110, NM_002745, NM_002086, NM_014247, NM_004586, NM_005921, NM_001130916, NM_006609, NM_002730	211	370	13528	2.25265	1	0.33501	19.6278
GOTERM_BP_FAT	GO:0006865~amino acid transport	6	2.07612	0.01302	NM_020949, NM_002111, NM_001145144, NM_020755, NM_012244, NM_001031681	211	90	13528	4.27425	1	0.33244	19.7903
GOTERM_BP_FAT	GO:0051247~positive regulation of protein metabolic process	10	3.46021	0.0137	NM_000208, NM_053056, NM_018724, NM_001759, NM_001161576, NM_003183, NM_000801, NM_001130916, NM_002745, NM_152756	211	243	13528	2.63843	1	0.3414	20.7145
GOTERM_BP_FAT	GO:0007569~cell aging	4	1.38408	0.01429	NM_001114979, NM_020338, NM_002111, NM_002524	211	33	13528	7.77136	1	0.34806	21.5012
GOTERM_BP_FAT	GO:0030838~positive regulation of actin filament polymerization	3	1.03806	0.01429	NM_006779, NM_005921, NM_152756	211	12	13528	16.0284	1	0.3432	21.5047
GOTERM_BP_FAT	GO:0043009~chordate embryonic development	12	4.15225	0.01451	NM_001017371, NM_020338, NM_002111, NM_003482, NM_030661, NM_000264, NM_001166283, NM_001130916, NM_002359, NM_001105077, NM_001145426, NM_004302	211	331	13528	2.32437	1	0.34269	21.8022
GOTERM_BP_FAT	GO:0008064~regulation of actin polymerization or depolymerization	5	1.7301	0.01478	NM_005720, NM_006779, NM_005921, NM_005717, NM_152756	211	61	13528	5.25522	1	0.34318	22.1674
GOTERM_BP_FAT	GO:0045941~positive regulation of transcription	17	5.88235	0.01482	NM_002957, NM_004423, NM_020338, NM_003206, NM_000333, NM_002745, NM_004555, NM_001001342, NM_001083962, NM_001114979, NM_001126054, NM_001142418, NM_005921, NM_001130916, NM_001098833, NM_001079846, NM_001105077	211	564	13528	1.93251	1	0.33916	22.2102
GOTERM_BP_FAT	GO:0009792~embryonic development ending in birth or egg hatching	12	4.15225	0.01524	NM_001017371, NM_020338, NM_002111, NM_003482, NM_030661, NM_000264, NM_001166283, NM_001130916, NM_002359, NM_001105077, NM_001145426, NM_004302	211	334	13528	2.30349	1	0.34238	22.7682
GOTERM_BP_FAT	GO:0006793~phosphorus metabolic process	25	8.65052	0.01623	NM_000208, NM_001258, NM_053056, NM_001396, NM_001042599, NM_002709, NM_020791, NM_004586, NM_001130916, NM_004302, NM_001143984, NM_003010, NM_002648, NM_002649, NM_015981, NM_002745, NM_016156, NM_003913, NM_001126054, NM_080836, NM_005921, NM_006609, NM_002710, NM_152835, NM_002730	211	973	13528	1.64732	1	0.35554	24.0624
GOTERM_BP_FAT	GO:0006796~phosphate metabolic process	25	8.65052	0.01623	NM_000208, NM_001258, NM_053056, NM_001396, NM_001042599, NM_002709, NM_020791, NM_004586, NM_001130916, NM_004302, NM_001143984, NM_003010, NM_002648, NM_002649, NM_015981, NM_002745, NM_016156, NM_003913, NM_001126054, NM_080836, NM_005921, NM_006609, NM_002710, NM_152835, NM_002730	211	973	13528	1.64732	1	0.35554	24.0624
GOTERM_BP_FAT	GO:0030334~regulation of cell migration	8	2.76817	0.01629	NM_000208, NM_005544, NM_001042599, NM_003183, NM_001087, NM_005921, NM_002745, NM_000214	211	169	13528	3.03497	1	0.35217	24.1469
GOTERM_BP_FAT	GO:0030832~regulation of actin filament length	5	1.7301	0.01648	NM_005720, NM_006779, NM_005921, NM_005717, NM_152756	211	63	13528	5.08839	1	0.35097	24.3885
GOTERM_BP_FAT	GO:0031399~regulation of protein modification process	11	3.80623	0.017	NM_000208, NM_053056, NM_018724, NM_001759, NM_003183, NM_000801, NM_005921, NM_001130916, NM_006609, NM_152756, NM_006761	211	295	13528	2.39068	1	0.35551	25.0649
GOTERM_BP_FAT	GO:0045935~positive regulation of nucleobase, nucleoside, nucleotide and nucleic acid metabolic process	18	6.22837	0.01749	NM_000208, NM_002957, NM_004423, NM_020338, NM_003206, NM_000333, NM_002745, NM_004555, NM_001001342, NM_001083962, NM_001114979, NM_001126054, NM_001142418, NM_005921, NM_001130916, NM_001098833, NM_001079846, NM_001105077	211	624	13528	1.84943	1	0.3592	25.6816
GOTERM_BP_FAT	GO:0040017~positive regulation of locomotion	6	2.07612	0.01823	NM_000208, NM_005544, NM_001042599, NM_003183, NM_001087, NM_002745	211	98	13528	3.92533	1	0.367	26.6262
GOTERM_BP_FAT	GO:0010628~positive regulation of gene expression	17	5.88235	0.01905	NM_002957, NM_004423, NM_020338, NM_003206, NM_000333, NM_002745, NM_004555, NM_001001342, NM_001083962, NM_001114979, NM_001126054, NM_001142418, NM_005921, NM_001130916, NM_001098833, NM_001079846, NM_001105077	211	581	13528	1.87596	1	0.37566	27.6528
GOTERM_BP_FAT	GO:0032885~regulation of polysaccharide biosynthetic process	3	1.03806	0.0193	NM_000208, NM_005544, NM_002709	211	14	13528	13.7387	1	0.37522	27.9609
GOTERM_BP_FAT	GO:0010962~regulation of glucan biosynthetic process	3	1.03806	0.0193	NM_000208, NM_005544, NM_002709	211	14	13528	13.7387	1	0.37522	27.9609
GOTERM_BP_FAT	GO:0045604~regulation of epidermal cell differentiation	3	1.03806	0.0193	NM_001114979, NM_018724, NM_002359	211	14	13528	13.7387	1	0.37522	27.9609
GOTERM_BP_FAT	GO:0005979~regulation of glycogen biosynthetic process	3	1.03806	0.0193	NM_000208, NM_005544, NM_002709	211	14	13528	13.7387	1	0.37522	27.9609
GOTERM_BP_FAT	GO:0001701~in utero embryonic development	8	2.76817	0.01989	NM_001017371, NM_020338, NM_003482, NM_001130916, NM_002359, NM_001105077, NM_001145426, NM_004302	211	176	13528	2.91426	1	0.37982	28.6764
GOTERM_BP_FAT	GO:0006417~regulation of translation	7	2.42215	0.02001	NM_001134649, NM_001130678, NM_001079533, NM_001161576, NM_002745, NM_006775, NM_145808	211	137	13528	3.27588	1	0.37745	28.8252
GOTERM_BP_FAT	GO:0002009~morphogenesis of an epithelium	6	2.07612	0.0205	NM_001114979, NM_003206, NM_002111, NM_000264, NM_001166283, NM_000214	211	101	13528	3.80874	1	0.38054	29.4213
GOTERM_BP_FAT	GO:0032271~regulation of protein polymerization	5	1.7301	0.02123	NM_005720, NM_006779, NM_005921, NM_005717, NM_152756	211	68	13528	4.71425	1	0.38702	30.3044
GOTERM_BP_FAT	GO:0032869~cellular response to insulin stimulus	5	1.7301	0.02123	NM_000208, NM_002957, NM_005544, NM_001759, NM_002086	211	68	13528	4.71425	1	0.38702	30.3044
GOTERM_BP_FAT	GO:0035295~tube development	9	3.11419	0.02164	NM_001017371, NM_032682, NM_001114979, NM_003206, NM_031476, NM_000264, NM_001166283, NM_001130916, NM_003873	211	220	13528	2.62283	1	0.38873	30.7929
GOTERM_BP_FAT	GO:0032881~regulation of polysaccharide metabolic process	3	1.03806	0.02205	NM_000208, NM_005544, NM_002709	211	15	13528	12.8227	1	0.3904	31.2792
GOTERM_BP_FAT	GO:0051173~positive regulation of nitrogen compound metabolic process	18	6.22837	0.02296	NM_000208, NM_002957, NM_004423, NM_020338, NM_003206, NM_000333, NM_002745, NM_004555, NM_001001342, NM_001083962, NM_001114979, NM_001126054, NM_001142418, NM_005921, NM_001130916, NM_001098833, NM_001079846, NM_001105077	211	644	13528	1.792	1	0.39881	32.3475
GOTERM_BP_FAT	GO:0016310~phosphorylation	21	7.26644	0.024	NM_000208, NM_001258, NM_001396, NM_053056, NM_001042599, NM_003010, NM_002648, NM_015981, NM_002649, NM_002745, NM_003913, NM_001126054, NM_020791, NM_080836, NM_004586, NM_005921, NM_001130916, NM_006609, NM_152835, NM_002730, NM_004302	211	800	13528	1.68299	1	0.40865	33.5531
GOTERM_BP_FAT	GO:0031401~positive regulation of protein modification process	8	2.76817	0.02657	NM_000208, NM_053056, NM_018724, NM_001759, NM_003183, NM_000801, NM_001130916, NM_152756	211	187	13528	2.74283	1	0.43724	36.437
GOTERM_BP_FAT	GO:0003007~heart morphogenesis	5	1.7301	0.02674	NM_000208, NM_002957, NM_020338, NM_000801, NM_000264	211	73	13528	4.39135	1	0.43519	36.6213
GOTERM_BP_FAT	GO:0016568~chromatin modification	10	3.46021	0.02745	NM_022756, NM_000333, NM_001142418, NM_003482, NM_014292, NM_006791, NM_001098426, NM_001098833, NM_001079846, NM_004100	211	274	13528	2.33992	1	0.43966	37.3902
GOTERM_BP_FAT	GO:0051896~regulation of protein kinase B signaling cascade	3	1.03806	0.02799	NM_000208, NM_001130916, NM_152756	211	17	13528	11.3142	1	0.44203	37.9711
GOTERM_BP_FAT	GO:0002053~positive regulation of mesenchymal cell proliferation	3	1.03806	0.02799	NM_032682, NM_001114979, NM_005544	211	17	13528	11.3142	1	0.44203	37.9711
GOTERM_BP_FAT	GO:0009725~response to hormone stimulus	12	4.15225	0.02824	NM_000208, NM_002957, NM_053056, NM_005544, NM_001042599, NM_001759, NM_000264, NM_001130916, NM_002745, NM_002086, NM_001014797, NM_002730	211	367	13528	2.09636	1	0.44097	38.2387
GOTERM_BP_FAT	GO:0009628~response to abiotic stimulus	12	4.15225	0.02839	NM_001018058, NM_002111, NM_053056, NM_001008224, NM_000264, NM_005921, NM_000684, NM_001130916, NM_000859, NM_001014797, NM_002524, NM_001145426	211	368	13528	2.09067	1	0.4388	38.4007
GOTERM_BP_FAT	GO:0040012~regulation of locomotion	8	2.76817	0.0301	NM_000208, NM_005544, NM_001042599, NM_003183, NM_001087, NM_005921, NM_002745, NM_000214	211	192	13528	2.67141	1	0.45439	40.2062
GOTERM_BP_FAT	GO:0010464~regulation of mesenchymal cell proliferation	3	1.03806	0.03117	NM_032682, NM_001114979, NM_005544	211	18	13528	10.6856	1	0.46217	41.3
GOTERM_BP_FAT	GO:0045893~positive regulation of transcription, DNA-dependent	14	4.84429	0.03525	NM_002957, NM_004423, NM_003206, NM_020338, NM_000333, NM_004555, NM_001083962, NM_001114979, NM_001126054, NM_001142418, NM_005921, NM_001098833, NM_001079846, NM_001105077	211	477	13528	1.88175	1	0.5008	45.3252
GOTERM_BP_FAT	GO:0015837~amine transport	6	2.07612	0.03677	NM_020949, NM_002111, NM_001145144, NM_020755, NM_012244, NM_001031681	211	118	13528	3.26002	1	0.51181	46.7614
GOTERM_BP_FAT	GO:0051254~positive regulation of RNA metabolic process	14	4.84429	0.03777	NM_002957, NM_004423, NM_003206, NM_020338, NM_000333, NM_004555, NM_001083962, NM_001114979, NM_001126054, NM_001142418, NM_005921, NM_001098833, NM_001079846, NM_001105077	211	481	13528	1.8661	1	0.51734	47.6792
GOTERM_BP_FAT	GO:0010563~negative regulation of phosphorus metabolic process	4	1.38408	0.03827	NM_000208, NM_000333, NM_000801, NM_006761	211	48	13528	5.34281	1	0.5181	48.1366
GOTERM_BP_FAT	GO:0045936~negative regulation of phosphate metabolic process	4	1.38408	0.03827	NM_000208, NM_000333, NM_000801, NM_006761	211	48	13528	5.34281	1	0.5181	48.1366
GOTERM_BP_FAT	GO:0042692~muscle cell differentiation	6	2.07612	0.04028	NM_032682, NM_002957, NM_006775, NM_145808, NM_000214, NM_002524	211	121	13528	3.17919	1	0.53265	49.9326
GOTERM_BP_FAT	GO:0040008~regulation of growth	11	3.80623	0.04036	NM_000208, NM_001114979, NM_001142418, NM_003183, NM_006791, NM_000264, NM_000684, NM_001130916, NM_003873, NM_001145426, NM_004302	211	341	13528	2.06819	1	0.52945	50.004
GOTERM_BP_FAT	GO:0007167~enzyme linked receptor protein signaling pathway	11	3.80623	0.04053	NM_000208, NM_005544, NM_001042599, NM_003183, NM_000801, NM_018110, NM_005921, NM_001130916, NM_002086, NM_003873, NM_004302	211	342	13528	2.06214	1	0.52706	50.1492
GOTERM_BP_FAT	GO:0043255~regulation of carbohydrate biosynthetic process	3	1.03806	0.0415	NM_000208, NM_005544, NM_002709	211	21	13528	9.15911	1	0.53182	50.9929
GOTERM_BP_FAT	GO:0015804~neutral amino acid transport	3	1.03806	0.0415	NM_001145144, NM_020755, NM_012244	211	21	13528	9.15911	1	0.53182	50.9929
GOTERM_BP_FAT	GO:0010828~positive regulation of glucose transport	3	1.03806	0.0452	NM_000208, NM_005544, NM_001042599	211	22	13528	8.74278	1	0.55923	54.0752
GOTERM_BP_FAT	GO:0046326~positive regulation of glucose import	3	1.03806	0.0452	NM_000208, NM_005544, NM_001042599	211	22	13528	8.74278	1	0.55923	54.0752
GOTERM_BP_FAT	GO:0045682~regulation of epidermis development	3	1.03806	0.0452	NM_001114979, NM_018724, NM_002359	211	22	13528	8.74278	1	0.55923	54.0752
GOTERM_BP_FAT	GO:0048608~reproductive structure development	6	2.07612	0.04657	NM_001114979, NM_003206, NM_053056, NM_001759, NM_000859, NM_001145426	211	126	13528	3.05304	1	0.56649	55.1744
GOTERM_BP_FAT	GO:0010608~posttranscriptional regulation of gene expression	8	2.76817	0.04666	NM_001134649, NM_001130678, NM_001079533, NM_001161576, NM_001018067, NM_002745, NM_006775, NM_145808	211	211	13528	2.43085	1	0.56336	55.2409
GOTERM_BP_FAT	GO:0007264~small GTPase mediated signal transduction	10	3.46021	0.04866	NM_152545, NM_004161, NM_014247, NM_005737, NM_002745, NM_198686, NM_002086, NM_002524, NM_001079874, NM_012425	211	305	13528	2.10209	1	0.57523	56.7963
GOTERM_BP_FAT	GO:0001889~liver development	4	1.38408	0.04903	NM_001017371, NM_002957, NM_053056, NM_001759	211	53	13528	4.83877	1	0.57434	57.0803
GOTERM_BP_FAT	GO:0032956~regulation of actin cytoskeleton organization	5	1.7301	0.04968	NM_005720, NM_006779, NM_005921, NM_005717, NM_152756	211	89	13528	3.6019	1	0.5756	57.5745

Supplementary table 4

Enriched BIOCARTA pathways	Count	%	p-value	Benjamini
MAPKinase Signaling Pathway	8	2.8	3.90E-03	4.00E-01
p38 MAPK Signaling Pathway	5	1.7	9.80E-03	4.80E-01
Links between Pyk2 and Map Kinases	4	1.4	2.90E-02	7.30E-01
Angiotensin II mediated activation of JNK Pathway via Pyk2 dependent signaling	4	1.4	3.20E-02	6.60E-01
Fc Epsilon Receptor I Signaling in Mast Cells	4	1.4	4.20E-02	6.80E-01
Synaptic Proteins at the Synaptic Junction	3	1	8.00E-02	8.40E-01
Multiple antiapoptotic pathways from IGF-1R signaling lead to BAD phosphorylation	3	1	8.00E-02	8.40E-01
Insulin Signaling Pathway	3	1	8.80E-02	8.20E-01

Enriched KEGG pathways	Count	%	p-value	Benjamini
Long-term potentiation	9	3.1	3.20E-05	3.30E-03
GnRH signaling pathway	10	3.5	7.60E-05	3.90E-03
Neurotrophin signaling pathway	11	3.8	9.00E-05	3.10E-03
Insulin signaling pathway	11	3.8	1.80E-04	4.70E-03
Oocyte meiosis	10	3.5	1.90E-04	3.80E-03
MAPK signaling pathway	15	5.2	3.80E-04	6.50E-03
Chronic myeloid leukemia	8	2.8	4.50E-04	6.60E-03
Non-small cell lung cancer	7	2.4	4.50E-04	5.80E-03
Glioma	7	2.4	1.00E-03	1.20E-02
B cell receptor signaling pathway	7	2.4	2.60E-03	2.60E-02
Endometrial cancer	6	2.1	2.70E-03	2.50E-02
Pathways in cancer	15	5.2	2.80E-03	2.40E-02
Fc epsilon RI signaling pathway	7	2.4	3.20E-03	2.50E-02
Acute myeloid leukemia	6	2.1	4.30E-03	3.10E-02
Colorectal cancer	7	2.4	4.60E-03	3.10E-02
ErbB signaling pathway	7	2.4	5.40E-03	3.40E-02
Aldosterone-regulated sodium reabsorption	5	1.7	6.80E-03	4.10E-02
Endocytosis	10	3.5	7.00E-03	4.00E-02
Fc gamma R-mediated phagocytosis	7	2.4	8.30E-03	4.40E-02
Melanogenesis	7	2.4	1.00E-02	5.10E-02
Adherens junction	6	2.1	1.40E-02	6.70E-02
mTOR signaling pathway	5	1.7	1.60E-02	7.10E-02
Thyroid cancer	4	1.4	1.60E-02	7.10E-02
Vascular smooth muscle contraction	7	2.4	1.80E-02	7.40E-02
Progesterone-mediated oocyte maturation	6	2.1	2.20E-02	8.60E-02
Gap junction	6	2.1	2.50E-02	9.50E-02
Prostate cancer	6	2.1	2.50E-02	9.50E-02
Jak-STAT signaling pathway	8	2.8	2.50E-02	9.20E-02
Renal cell carcinoma	5	1.7	4.10E-02	1.40E-01
Pancreatic cancer	5	1.7	4.50E-02	1.50E-01
Regulation of actin cytoskeleton	9	3.1	4.70E-02	1.50E-01
T cell receptor signaling pathway	6	2.1	5.10E-02	1.60E-01
VEGF signaling pathway	5	1.7	5.10E-02	1.50E-01
Notch signaling pathway	4	1.4	5.70E-02	1.70E-01
Type II diabetes mellitus	4	1.4	5.70E-02	1.70E-01
Wnt signaling pathway	7	2.4	6.20E-02	1.80E-01
Dorso-ventral axis formation	3	1	7.80E-02	2.10E-01
Focal adhesion	8	2.8	8.10E-02	2.10E-01
RNA degradation	4	1.4	9.00E-02	2.30E-01

Supplementary figure 1

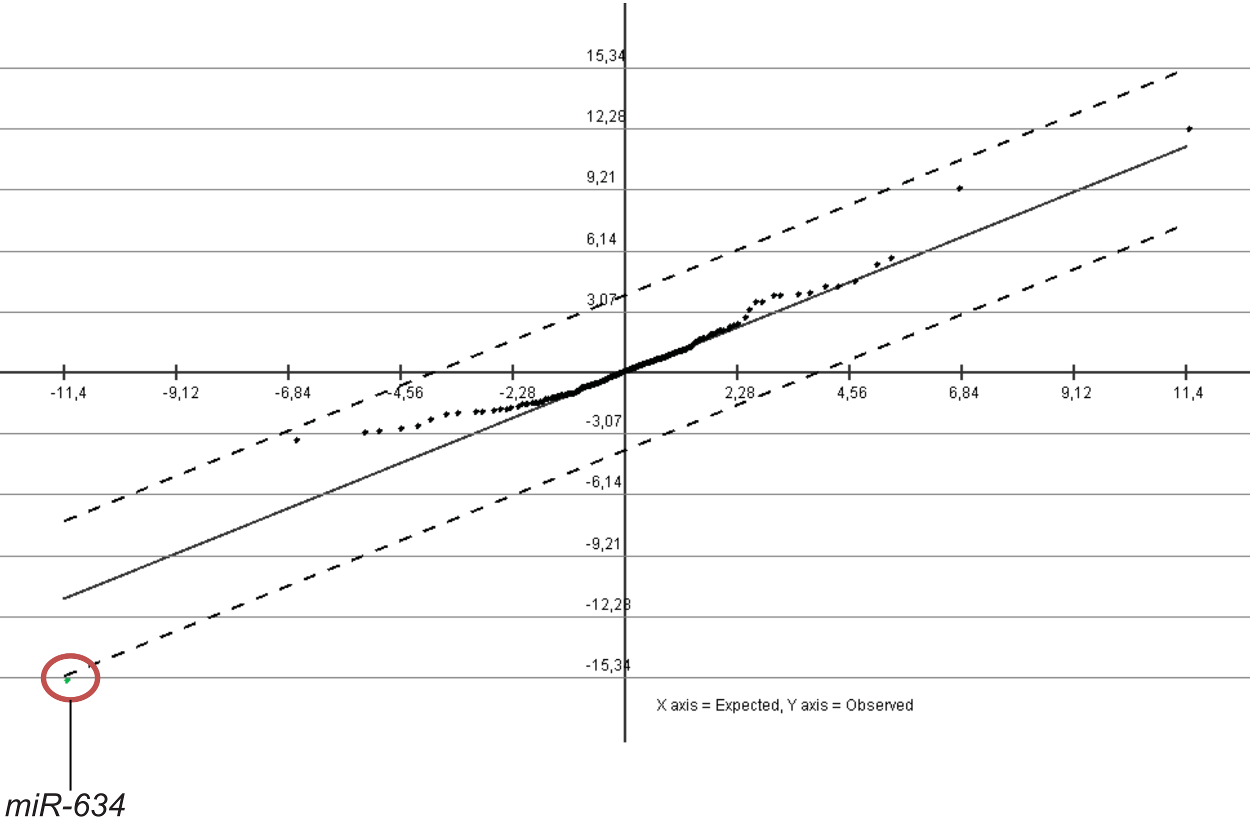

Supplementary figure 2

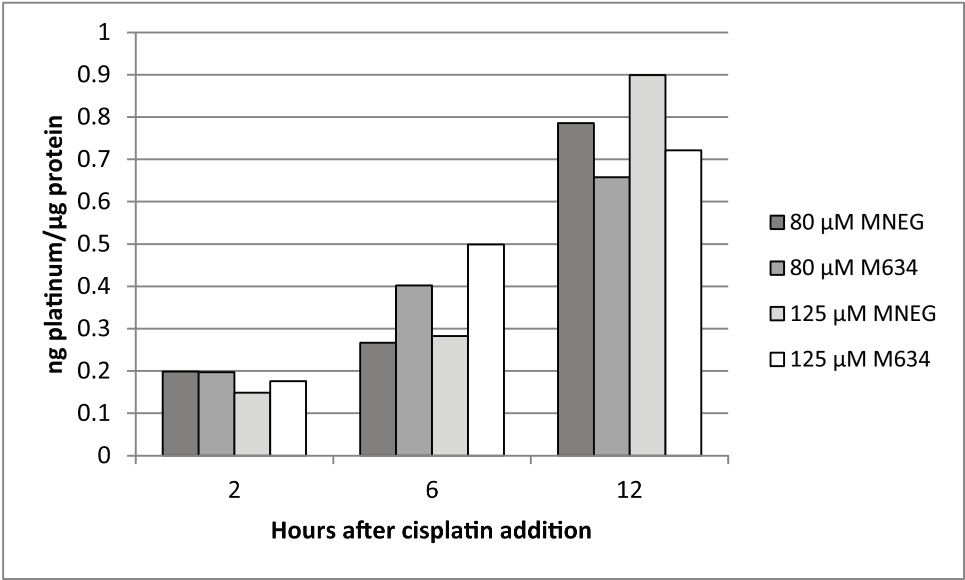

Supplementary figure 3

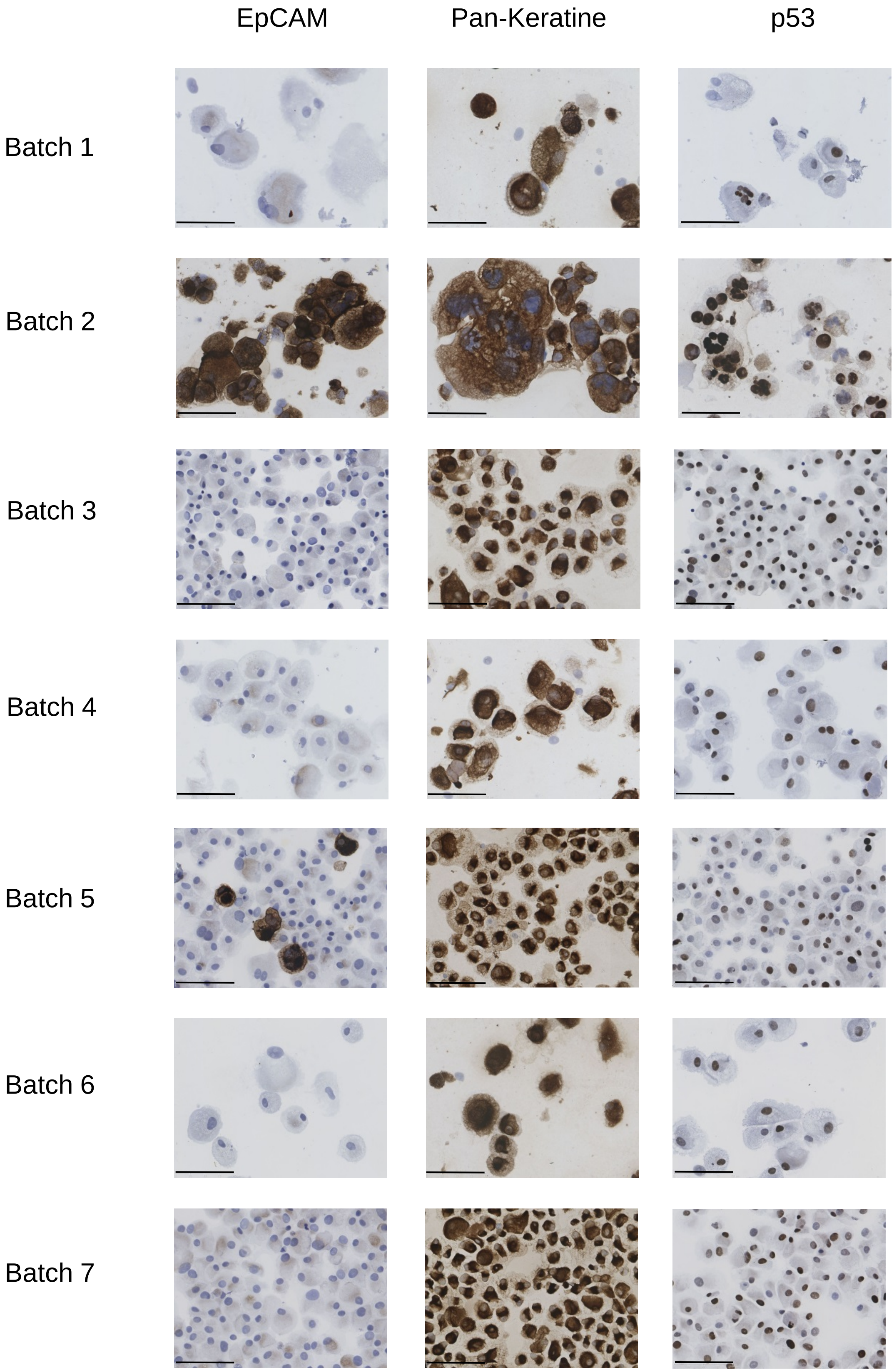

Supplementary figure 4

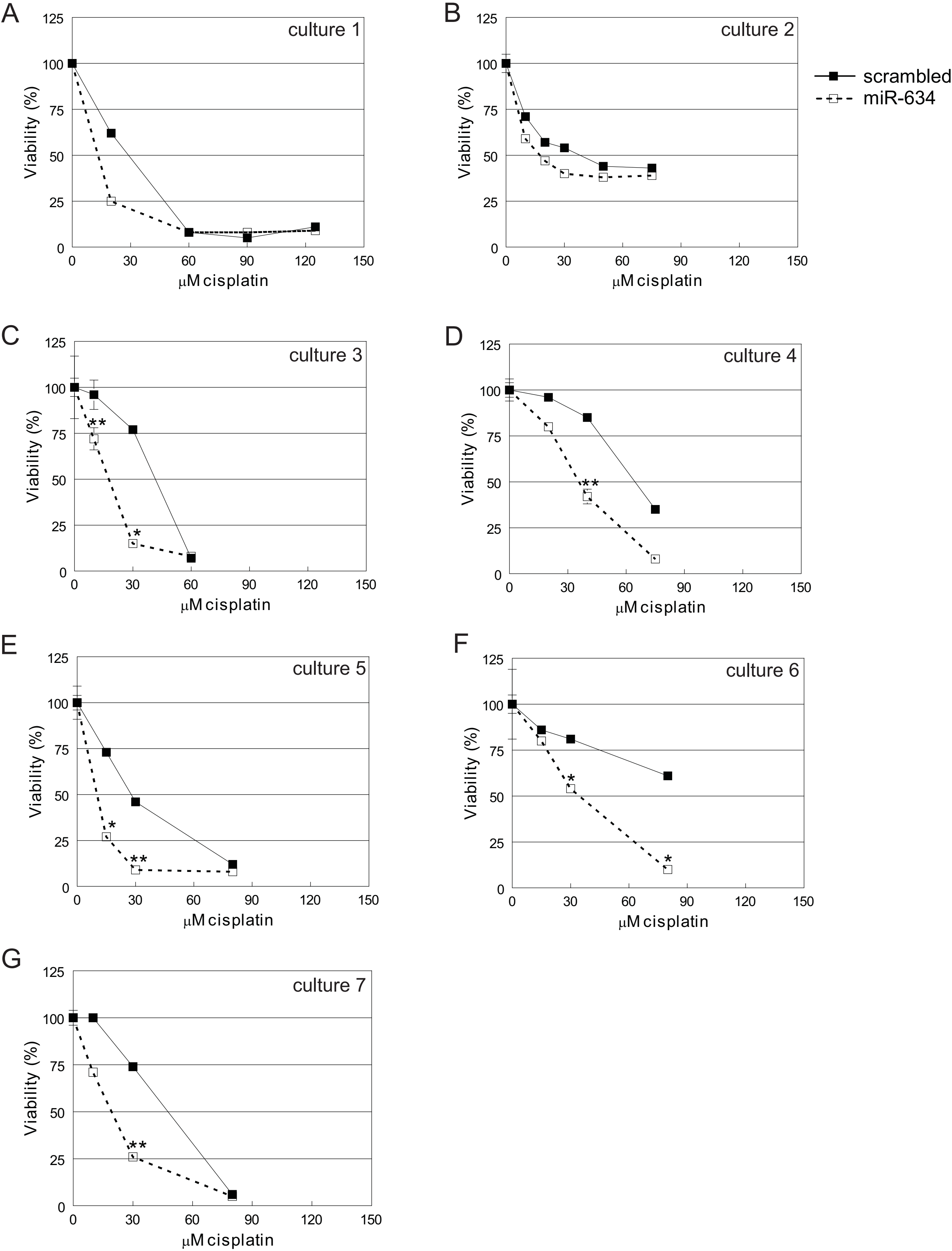

Supplementary figure 5

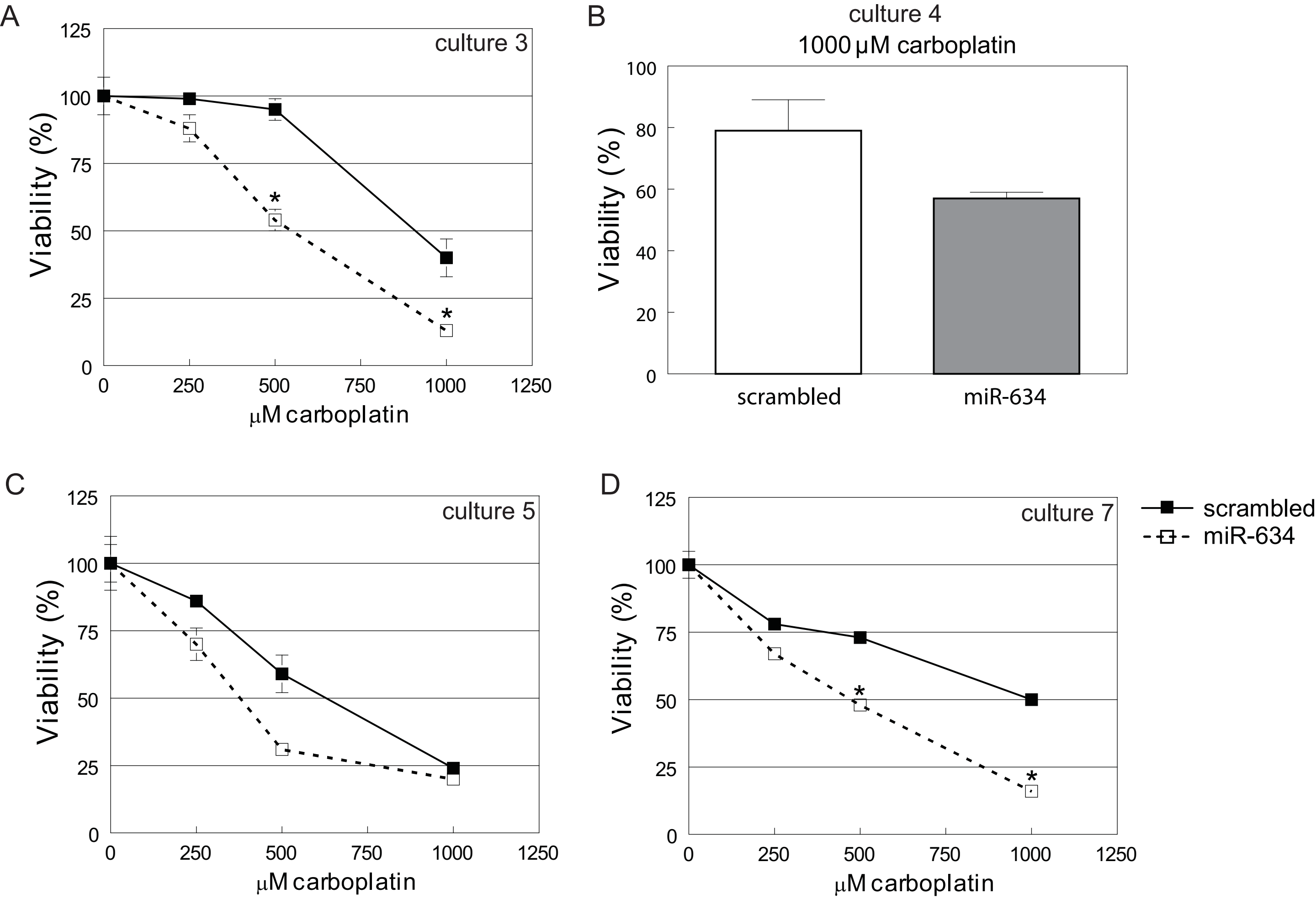

Supplementary figure 6

A

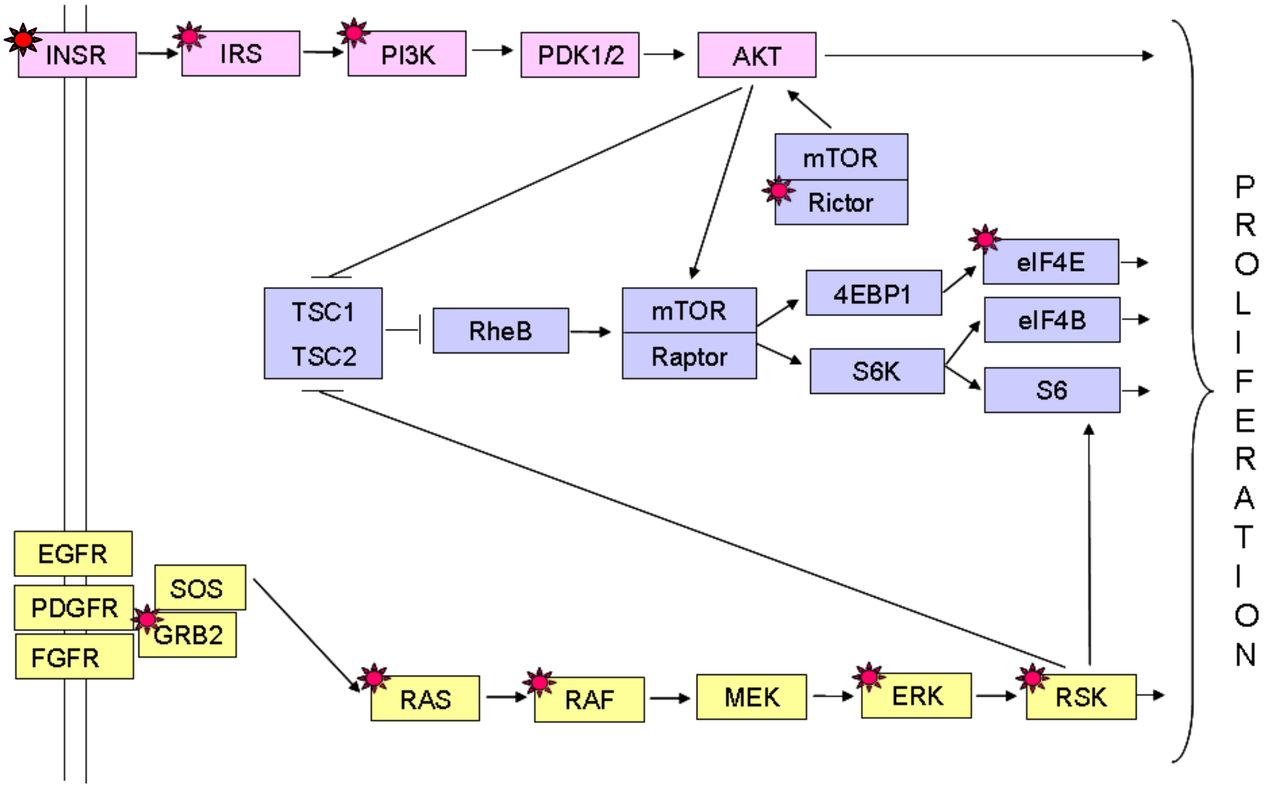

B

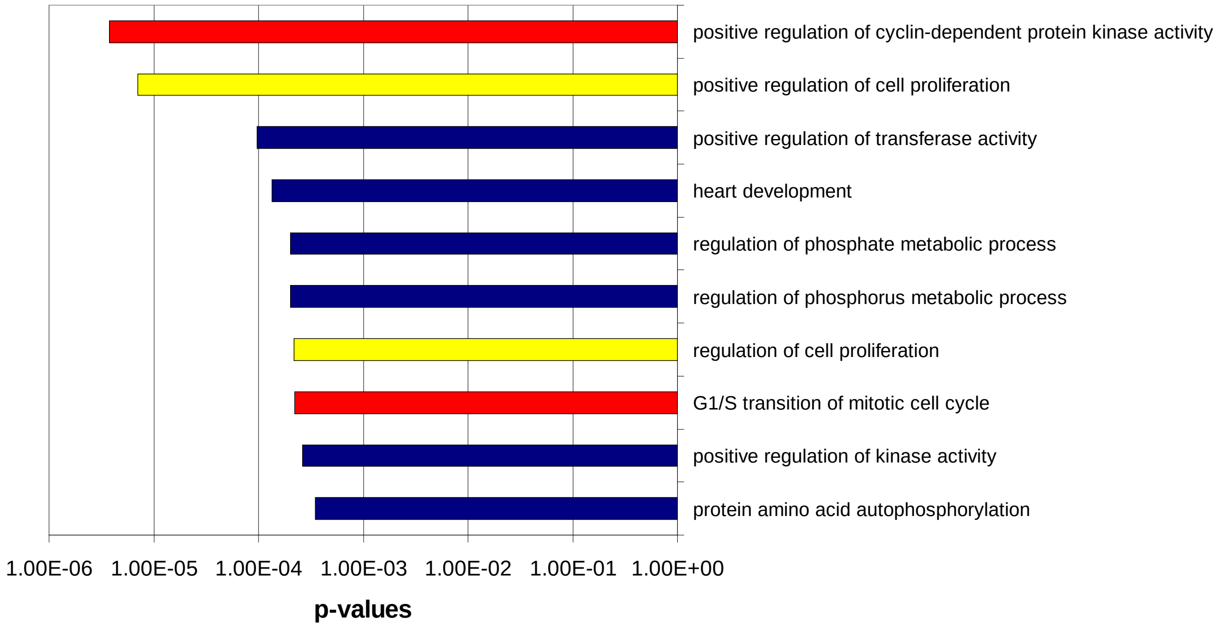

Supplementary figure 7

A

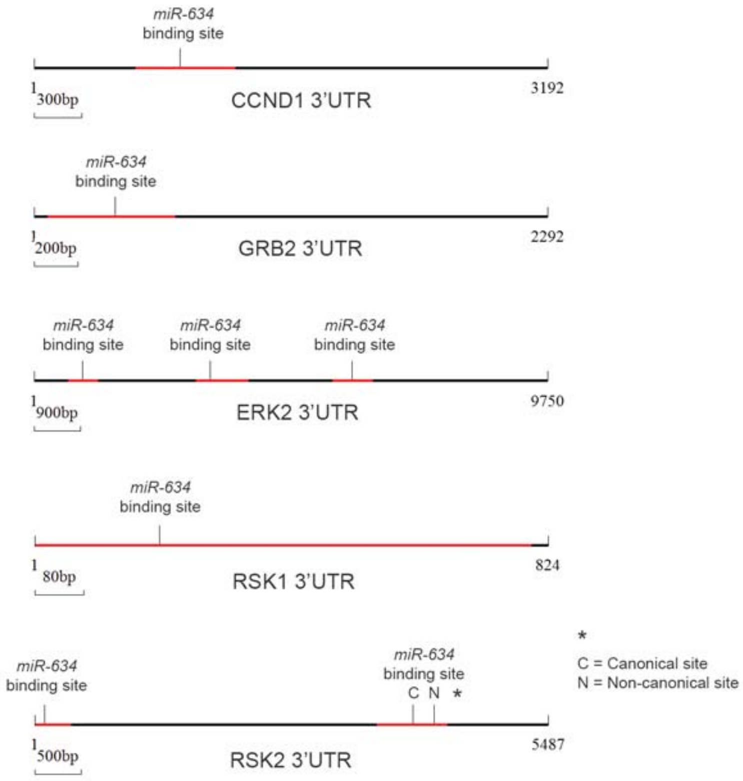

B

				FW primer		RV primer		Mutant	Mutant primer sense	Mutant primer antisense
	3'UTR length	Construct	position miR-634 site	position	sequence	position	sequence			
CCND1	3192	CCND1	908	632	5'CGCTCGAGCCTCTTCACCTTATTCATG	1229	5'ATGCGGCCGCGGAAAGGAACTTATCATCC	CCND1_mut	5'-gtgacctgtttatgagatcccgggtttctacccaacggccct-3	5'-agggccgttgggtagaaacccgggatctcataaacaggtcac-3'
GRB2	2292	GRB2	361	64	5'CGCTCGAGCACAAGCTGCCTCTGACAG	610	5'ATGCGGCCGCCAGGTGGGCTTTCTCAGG	GRB2_mut	5'-taataaaaaacaagaaaccaagtggcccgggattctctctatgcaaaatgtctgtt-3'	5'-aacagacattttgcatagagagaatcccgggccacttggtttcttgtttttatta-3'
ERK2	9750	ERK2_1	917	650	5'CGCTCGAGCCCAGCTTTTAGAAAATGC	1197	5'ATGCGGCCGCTCCAAACACGAGTTAGTGC	ERK2_1_mut	5'-cagggtggaactccacatcccgggcatatacgccc-3'	5'-gggcgtatatgcccgggatgtggagttccaccctg-3'
		ERK2_2	3352	3076	5'CGCTCGAGAGCCATTCAGAGGAAACTG	4045	5'ATGCGGCCGCCTCACAGCCCTAACACAAG	ERK2_2_mut	5'-tatacatttttaaagcatttgtgctttaaaattgttatcccgggtttgaaacatgatactcctg-3'	5'-caggagtatcatgtttcaaaccccgggataacaattttaaagcacaaatgctttaaaaatgtata-3'
		ERK2_3	6034	5670	5'CGCTCGAGCGGACAATGATATAGAGCG	6414	5'ATGCGGCCGCGGAACCCCACAGAAACATC			
RSK1	824	RSK1	201	1	5'CGCTCGAGGGCACCAGGGCATTCGG	773	5'ATGCGGCCGCCATGGACAGATGAACCCTGAGTAAC			
RSK2	5487	RSK2_1	106	1	5'CGCTCGAGAGTGACCTCAGTGAGATATTTG	369	5'ATGCGGCCGCGCTTAACATAAACTGTCCATCC	RSK2_1_mut	5'-ctgcaagcacacactgtcccacccgggacccataatgc-3'	5'-gcattatgggtcccgggtgggacagtgtgtgcttgcag-3'
		RSK2_2	Canonical 4043	3661	5'CGCTCGAGCTGGCCTTTAAAAATGACC	4391	5'ATGCGGCCGCCTCGGTATGCCCTGTTATG	RSK2_2_mut_1	5'-tcaaccccaggttgcttacattttcccgggctccccaagtgac-3'	5'-gtcacttggggagcccgggaaaatgtaagcaacctggggttga-3'
			Obscure 4267					RSK2_2_mut_2	5'-gtattctgtgtgatcaagtgatttgcacccgggtccaatgtgactgag-3'	5'-ctcagtcacattggacccgggtgcaaatcacttgatcacacagaatac-3'
					Indicated in red is the XhoI site that is added via PCR amplification		Indicated in red is the NotI site that is added via PCR amplification		Indicated in red is the miR-634 target site mutated to a SmaI site	Indicated in red is the miR-634 target site mutated to a SmaI site

Supplementary figure 8

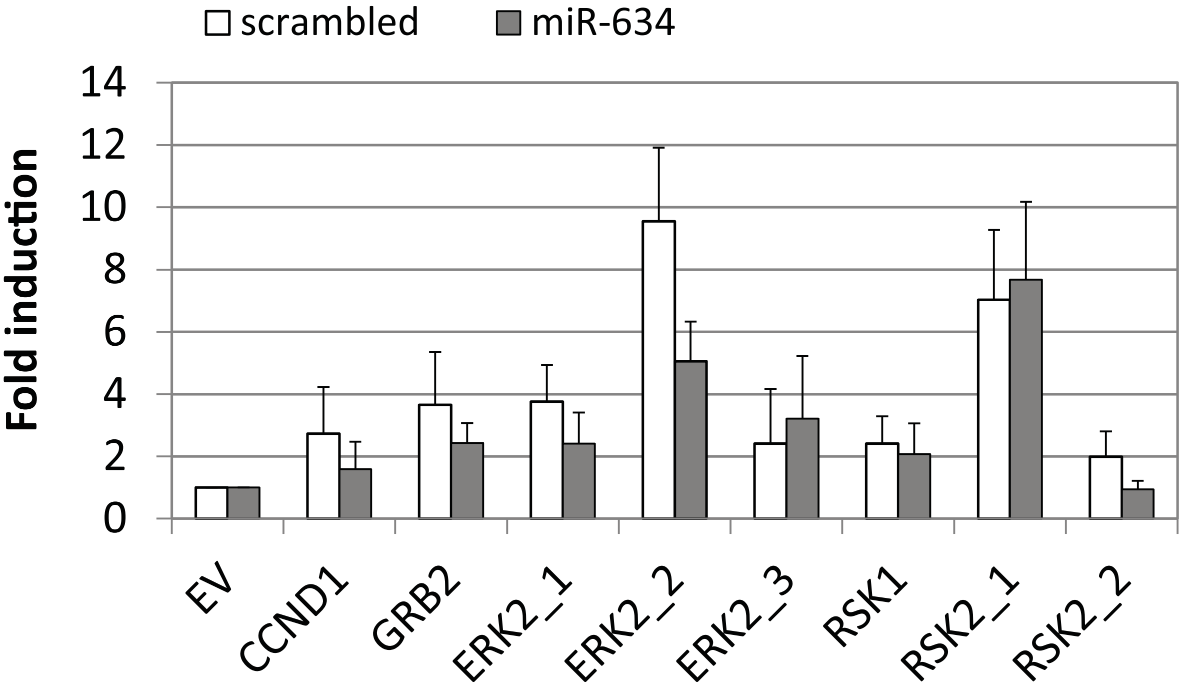

Supplementary figure 9

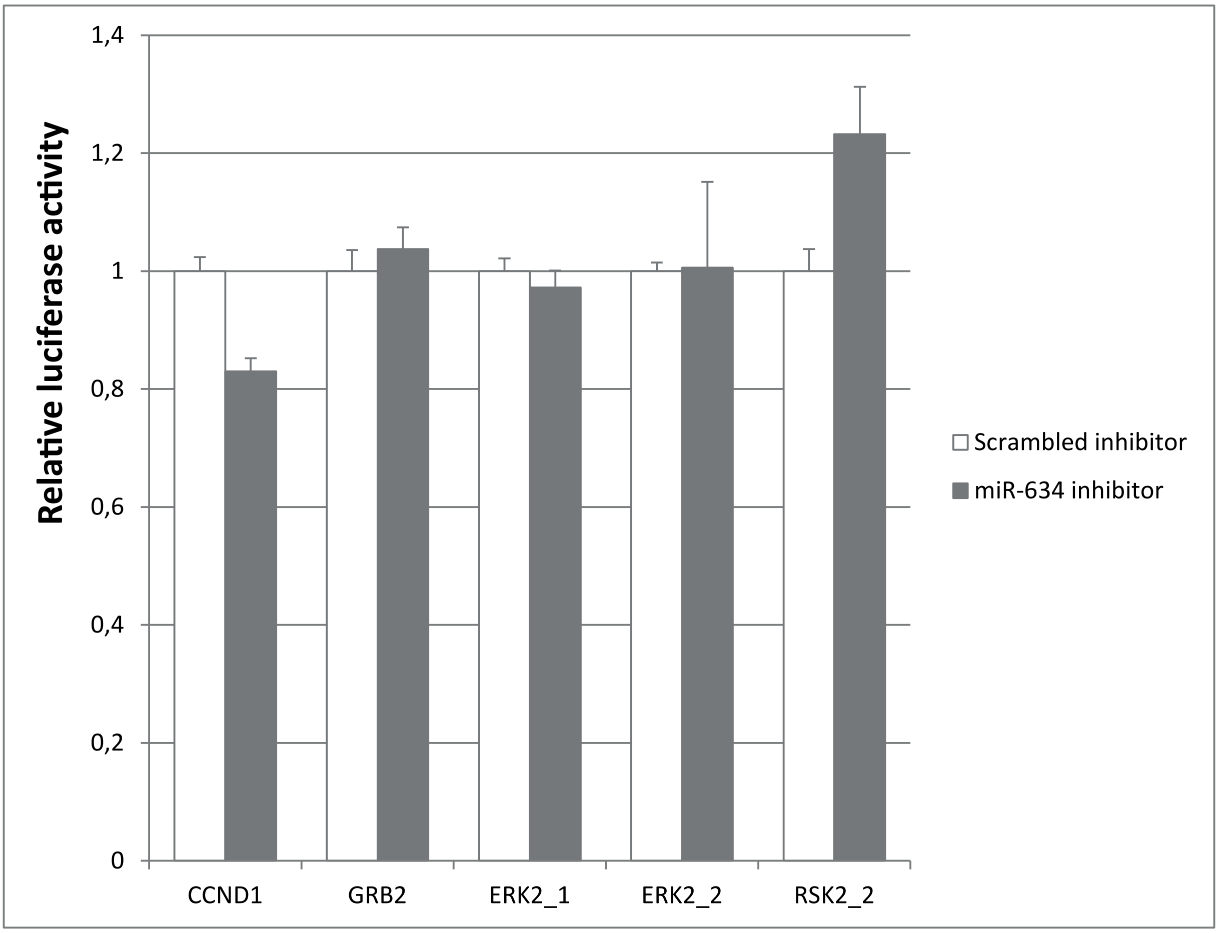

Supplementary figure 10

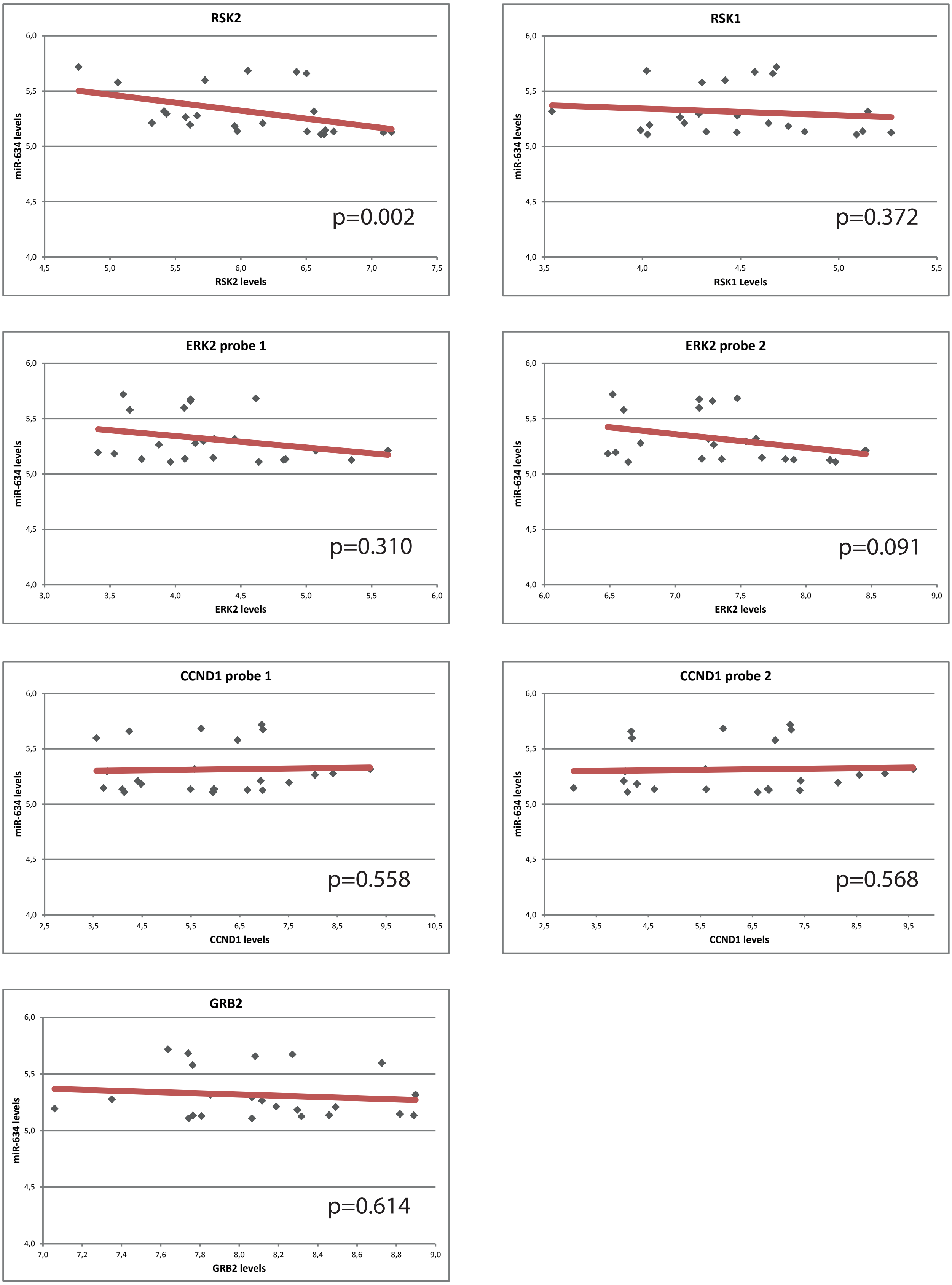

Supplementary figure 11

A

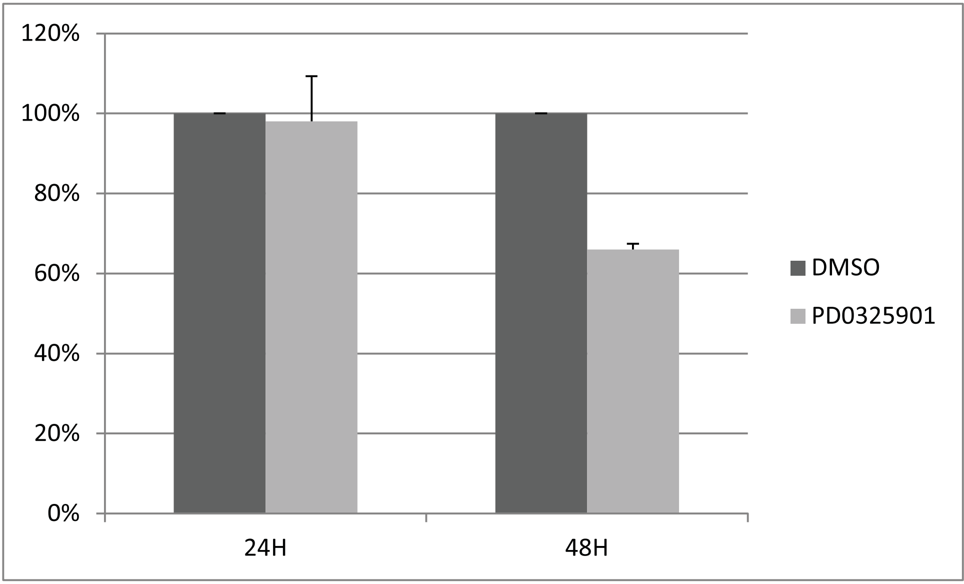

B

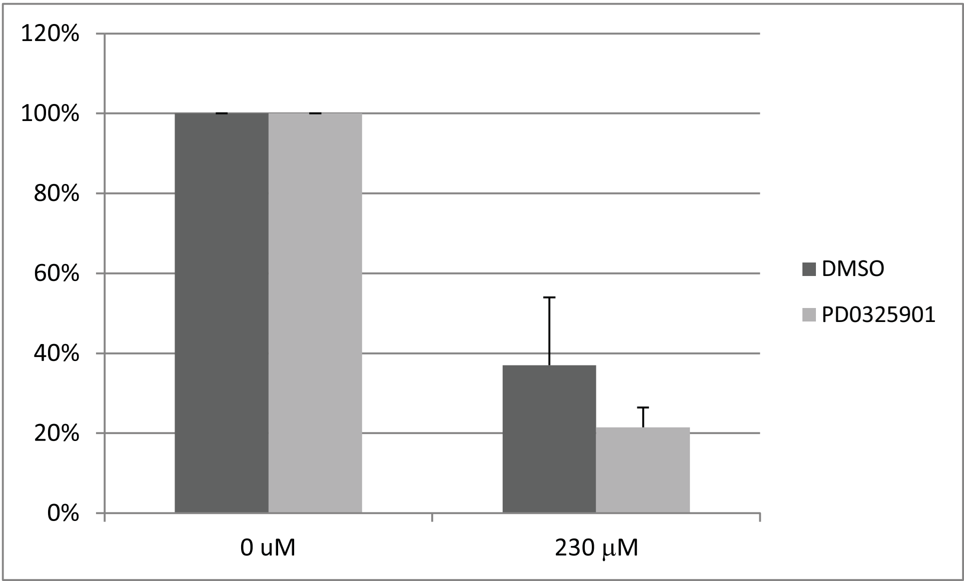

Supplementary figure 12

A.

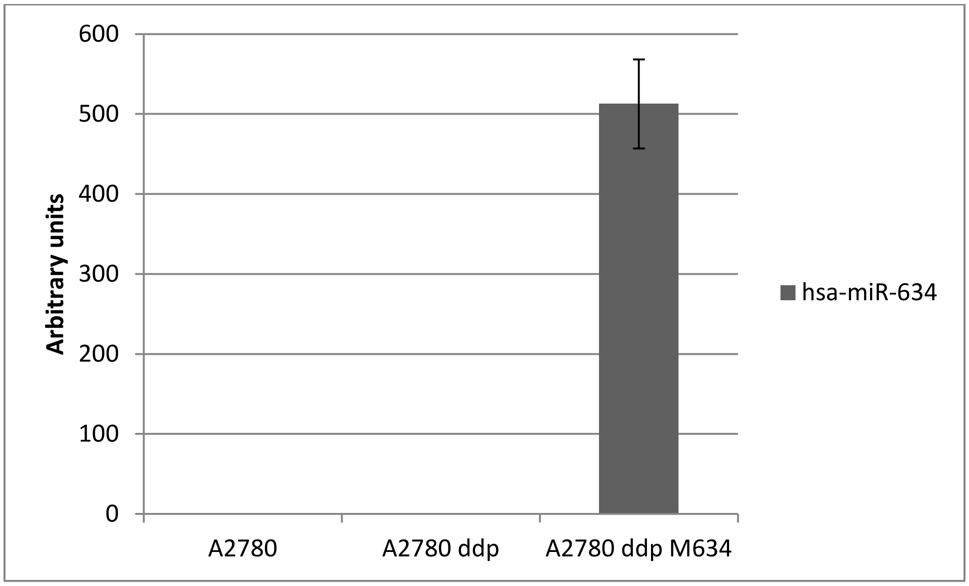

B.

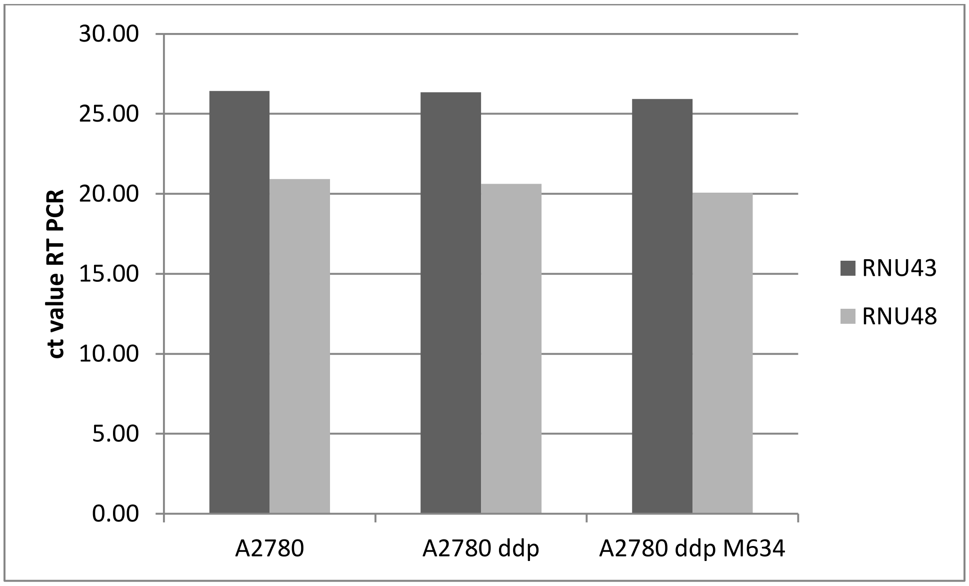

Supplement: Additional file 1: Table S1. — Cisplatin sensitivity of cell lines. Table S2. miRNAs that show a ≥1.5 fold change of expression in cisplatin sensitive and resistant cell lines. Table S3. GO-terms that are enriched for miR-634 targets. Table S4. An overview of KEGG and Biocarta pathways that are enriched for miR-634 targets. Figure S1. miR-634 is the only miRNA showing consistent changes in cisplatin sensitive and resistant cell lines. Figure S2. Platinum levels in A2780 DDP cells transfected with miR-634 or scrambled controls. Figure S3. Immunohistochemical staining of primary ovarian cancer cell cultures. Figure S4. miR-634 enhances cisplatin sensitivity. Figure S5. miR-634 enhances carboplatin sensitivity. Figure S6. Predicted miR-634 targets. Figure S7. Overview of the location of the miR-634 target sites in the 3’UTR of its potential target genes. Figure S8. Effect of miR-634 overexpression on luciferase activity of psiCHECK™-2 reporter constructs containing part of the 3’UTR of miR-634 predicted targets. Figure S9. Effect of miR-634 inhibitors on luciferase activity of psiCHECK™-2 reporter constructs containing part of the 3’UTR of miR-634 predicted targets. Figure S10. Correlation between miR-634 expression and its target gene expression in 23 miR-634 expressing primary ovarian cancer samples (TCGA dataset). Figure S11. Effect of PD0325901 on cell survival and cisplatin sensitivity. Figure S12. Detection of miR-634, RNU43 and RNU48 by RT-PCR. (PDF 1609 kb) [file 12943_2015_464_MOESM1_ESM.pdf]
